# Oral Health and Its Associated Factors Among Older Institutionalized Residents—A Systematic Review

**DOI:** 10.3390/ijerph16214132

**Published:** 2019-10-26

**Authors:** Florence M. F. Wong, Yannies T. Y. Ng, W. Keung Leung

**Affiliations:** 1 School of Nursing, Tung Wah College, Hong Kong SAR, China; 2 North District Hospital, Hospital Authority, Hong Kong SAR, China; Yannies.NTY@gmail.com; 3 Faculty of Dentistry, The University of Hong Kong, Hong Kong SAR, China; ewkleung@hku.hk

**Keywords:** aged, geriatric dentistry, health services for the aged, nursing homes, oral health, systematic review

## Abstract

The oral health of an ageing population, especially that of the institutionalized elderly population, constitutes a significant concern because it is closely linked to general health and the quality of life. Shared common risk factors drive the development and worsening of poor oral health and non-communicable diseases, which eventually lead to self-care inability. Several studies have reported on the poor oral health of the institutionalized elderly population. However, few comprehensive reports exist regarding the relationship between poor oral health, the oral health-related quality of life (OHRQoL) and the associated factors in this specific population. *Objective:* The objective is to describe recently reported oral health levels, the OHRQoL and the associated factors among older institutional residents. *Methods:* Studies published between July 2009 and June 2019 in MEDLINE, EMBASE and CINAHL were searched. The population, intervention, comparison and outcome (PICO) strategy was used as a guide. The reported factors related to poor oral health were identified (i.e., age, gender, educational level, acquired systemic conditions or dementia/cognitive impairment). *Results:* Twenty-five surveys (or study series) from 19 countries were included. The level of evidence reported by these studies was generally moderate to strong. The reported oral cleanliness and health of the surveyed institutionalized elderly were poor (>50% of residents had calculus; denture hygiene index > 80%). Gum (approximately 30% of dentate residents had moderate to severe periodontitis), teeth (decayed, missing or filled teeth >20), mucosa (>10% had mucosal lesions) and denture problems (up to 40%) were prevalent and were associated with a poor OHRQoL, especially in females, socially deprived residents or those with mild or above cognitive impairment. Those with a poor OHRQoL might show signs of poor nutrition. *Conclusions:* This report reviewed evidence-based knowledge on oral health, the OHRQoL and the associated factors among elderly institutional residents. Further research is needed to confirm these observations. For improved oral health, a better OHRQoL and the general well-being of older residents, clinical trials are needed, targeting modifiable factors, such as social inequality, oral healthcare accessibility, and/or nursing home service quality. The relationship between oral health, the OHRQoL and nutrition in this at-risk population also warrants exploration.

## 1. Introduction

Oral health is gaining global attention because it is closely linked to general health and the quality of life [1]. As the global population ages, healthcare services for elderly people have been further developed to improve their health and quality of life [2,3]. Among adults 65 years old or older, missing natural teeth and chronic oral diseases, such as dental caries, periodontal diseases, oral infections, oral mucosal lesions and temporomandibular disorders, are common [4]. Older people often suffer from chronic illnesses for which daily medications are needed. One common oral side effect of medications is hyposalivation [5,6]. Caries or mucosal infections dramatically increase with the impairment of the saliva function, giving rise to various oral health complications [5,6]. These problems make the fulfilment of basic daily needs (e.g., chewing and communication) more difficult, leading to consequential physical health problems, such as nutritional inadequacy and psychosocial distress (e.g., low self-esteem and social insufficiency) [7,8]. The institutionalized elderly population is a vulnerable subpopulation and is often care-dependent with poor oral health [7,8]. Numerous studies were published profiling the oral health and the oral health-related quality of life (OHRQoL) of the institutionalized elderly population, using validated instruments or approaches (Appendix 1).

Oral health is closely linked to the OHRQoL [9], which has become a significant patient-centered parameter for evaluating oral health outcomes. Self-perceived poor oral health predicts poor self-rated general health, self-esteem and life satisfaction, indicating the conscious and psychological link between oral health, general health and psychological well-being [10]. The OHRQoL tools evaluate the well-being of the surveyed elderly in terms of physical and psychosocial functioning in relation to orofacial concerns [11]. Validated and commonly used OHRQoL instruments targeting the elderly include the General or Geriatric Oral Health Assessment Index (GOHAI) [12], Oral Health Impacts Profile (OHIP) [13] and Oral Impact on Daily Performance (OIDP) [14] (Appendix 1). Based on the measurements from these OHRQoL instruments, previous studies have reported a relatively poor OHRQoL in the institutionalized elderly population due to their generally poor oral health [15,16].

Oral health can be determined by different factors among the elderly, including those who are institutionalized, especially those who have limited functional or self-care ability. As the corresponding awareness among healthcare providers attending institutionalized elderly populations increases, it is important for everyone involved to understand and comprehend the oral health challenges this specific vulnerable population faces. The risk factors associated with their poor oral health must be identified and appropriately intercepted. Proper oral health among the institutionalized elderly population around the world is not yet secured. Various studies have provided findings regarding the prevalence, effects and so on of common oral health problems of this at-risk group. The knowledge so far remains fragmented. Therefore, this systematic review aims to integrate the knowledge on the oral health status of the institutionalized elderly population through a comprehensive evaluation of the reported evidence over the past decade and to identify factors that might be associated with poor oral health and the OHRQoL. We anticipate that the outcomes of this review might inspire prevention protocols or ideas for better management of this at-risk minority population.

## 2. Materials and Methods 

### 2.1. Literature Search and Selection Process

The systematic procedure followed the item checklist using the Preferred Reporting Items for Systematic reviews and Meta-Analyses (PRISMA) statement [17,18] (Table S1). The initial literature search was conducted for both published and unpublished qualitative and quantitative studies published between July 2009 and June 2019 via the databases of the Cochrane Library, Joanna Briggs Institute (JBI) Library of Systematic Reviews, MEDLINE (OvidSP), EMBASE and CINAHL. Hand searches were performed on key journals, such as Gerodontology, the Journal of Periodontology, the Journal of Clinical Periodontology and the International Journal of Nursing Studies and the reference lists of all included studies. Google Scholar was also searched. The search strategy was based on keywords and medical subject heading (MeSH) terms. The keywords were used for searching for relevant studies related to the following:

(a) Oral health status:
  - Oral health, oral problems, Community Periodontal Index (CPI), loss of attachment, Community Periodontal Index of Treatment Needs (CPITN), Decayed Missing Filled Teeth (DMFT) Index, Denture Hygiene Index (DHI), Denture Plaque Index (DPI), Gingival Bleeding Index (GBI), Gingival Index for Long-Term Care (GI-LTC), Periodontal Screening and Recording (PSR), Revised Oral Assessment Guide (ROAG) and Visual Plaque Index (VPI).
  - Oral health-related quality of life (OHRQoL), General/Geriatric Oral Health Assessment Index (GOHAI), Oral Health Impacts Profile (OHIP) and Oral Impact on Daily Performance (OIDP).
(b) Target population—aged, elderly, older people and residents;
(c) Target setting—institutionalized homes, long-term care/old age homes, nursing homes and residential homes;
(d) Factors—factors, predictors, determinants and precipitating factors.

The searches via various databases were recorded in Tables S2–S4.

**Figure 1:**
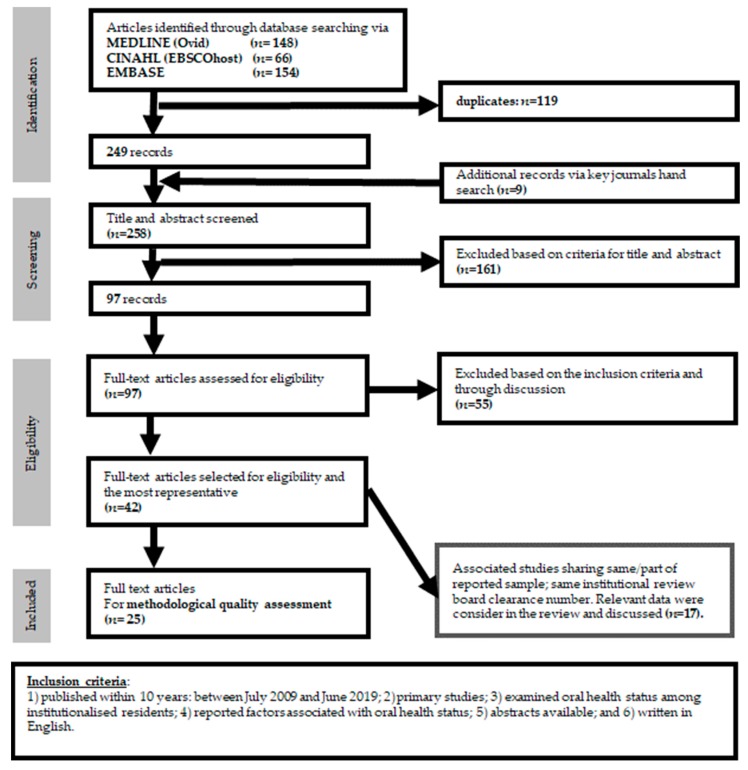
Preferred Reporting Items for Systematic Reviews and Meta-analysis (PRISMA) Flow Diagram: This figure shows the study selection process for included studies on oral health and its associated factors among institutionalized residents.

This systematic review was conducted following the Preferred Reporting Items for Systematic Reviews and Meta-analysis (PRISMA) guidelines [17,18]. To select relevant studies, the four-step PRISMA flow diagram (Figure 1) identification, screening, eligibility and included was followed by two independent reviewers (F.M.F.W and Y.T.Y.N). Then, the full texts of the selected studies were retrieved and two independent reviewers (F.M.F.W and Y.T.Y.N) reviewed the studies for data extraction, methodological quality assessment and analysis. All discrepancies were resolved through discussion or by consulting the third reviewer (WKL) until a consensus was reached. 

### 2.2. Inclusion and Exclusion Criteria

The search was limited to studies that (1) were published within the 10 years between July 2009 and June 2019, (2) were primary studies, (3) examined and reported the oral health status of the institutionalized elderly population, (4) reported factors associated with oral health status, (5) had available abstracts and (6) were written in English in the electronic databases. The following documents were excluded—(1) reviews (literature or systematic reviews), clinical guidelines or recommendations, editorials or reports of expert opinion and (2) studies conducted to validate assessment tools.

The PICO strategy [18], namely, population, intervention, comparison and outcomes, was used as a guide for retrieving the relevant articles for this review.

- Population (P): The target of this review was the institutionalized elderly population.
- Intervention (I): Studies with interventions to improve oral health as one of the factors were reviewed.
- Comparison (C): Studies with comparisons of oral health of the institutionalized elderly population with or without a specific oral or general condition were reviewed.
- Outcome (O): Studies that examined oral health and/or the oral health quality of life among the institutionalized elderly population that reported associated factors were reviewed.
- The included studies are listed under Appendix 2 and Table S5 shows details about the excluded studies.

### 2.3. Data Extraction and Data Analysis 

The data were extracted and recorded using the structured data extraction form adapted from the Cochrane Developmental, Psychosocial and Learning problems [19]. The form includes the authors, years of publication, titles, journals, aims/purposes, types of study, sampling (sample calculation, sampling method, inclusion and exclusion criteria and attrition rate), settings, instruments, analytical methods, main results (oral problems and the OHRQoL among the institutionalized elderly population and the associated factors), limitations and funding.

A thematic analysis of all included studies was conducted by two independent reviewers (F.M.F.W and Y.T.Y.N) to identify oral problems and assess the OHRQoL and the associated factors among the institutionalized elderly population. The findings were then compared. However, due to inadequate data for the meta-analysis from the included studies, a descriptive report was conducted.

### 2.4. Quality Assessment

Two reviewers (F.M.F.W and Y.T.Y.N) independently assessed the methodological quality of each included study based on the JBI critical appraisal tool to determine the extent to which an individual study addressed the possibility of bias in its methodological design, conduct and analysis [20]. All discrepancies were discussed between the two reviewers until a consensus was reached or a third reviewer (WKL) was consulted. 

## 3. Results

### 3.1. Study Selection 

A total of 377 articles were identified based on the keywords and MeSH terms searched for in the EMBASE (Ovid), CINAHL and MEDLINE (OvidSP) databases (Figure 1, Tables S2, S3, S4, respectively). After removing duplicates, 249 articles were identified. Another nine articles were identified after hand-searching key journals. The titles and abstracts of these articles were screened and 161 were excluded. These 97 articles were carefully scrutinized and 55 were excluded (Table S5) after agreement by both reviewers (F.M.F.W and Y.T.Y.N) with comments from the third reviewer (WKL) when required. Among the remaining 42 articles, 17 papers were related reports (i.e. sharing the same sample and hence the same institutional review board approval with the related article). Eventually, 25 studies/study series were identified to be eligible and were included in this review (Appendix 2 & Figure 1). Articles cited by and citing these 25 studies were reviewed using Google Scholar. No additional study was identified. 

### 3.2. Included Studies

#### 3.2.1. Methodological Quality 

Twenty-two of the included studies used a cross-sectional design [21,22,23,24,25,26,27,28,29,30,31,32,33,34,35,36,37,38,39,40,41,42] and three [43,44,45] used a case-control design or incorporated some sort of reference group. The JBI critical appraisal checklists for cross-sectional studies and case-control studies were applied to evaluate the methodological quality. Twelve cross-sectional studies were rated as strong, nine as moderate and one as weak (Table 1), whereas one case-control report was rated as strong and two as moderate (Table 2). Overall, the weaknesses of the included studies were primarily the small sample sizes, inadequate data reporting, inability to handle cofounding factors or poor control selection in the case-control study.

#### 3.2.2. Characteristics

Among the 25 studies/series (*n* = 10,958), the following were found:

- eight from Asia (*n* = 1851),
  ∘ three from India [21,22,23,46],
  ∘ two from Iran [24,25],
  ∘ one each from South Korea [43], Hong Kong [26] and Japan [27,47];
- two from the Eurasian trans-continent (*n* = 460),
  ∘ Turkey [28,29,48];
- eleven from Europe (*n* = 5899),
  ∘ one each from Belgium [32,49], Finland [33,50], Italy [34], Lithuania [44], Malta [35,51], the Netherlands [45,52], Spain [36] and the United Kingdom [37];
  ∘ two from Germany [30,31,53,54,55,56,57],
- two from Australia (*n* = 715) [38,39,58,59];
- two from North America (*n* = 841),
  ∘ one each from Canada [40,60] and the USA [41];
- one (*n* = 1192) from South America,
  ∘ Brazil [42,61]; but
- none from Africa.

Three studies included a community-dwelling control group [43,44,45,52] with conveniently sampled elderly participants, except the Lithuanian study, which included participants with a mean of 22.3 years of age. One Canadian study [40] included a community-dwelling group for the OHRQoL assessment without a detailed oral examination and was therefore considered a cross-sectional study. Table S6 provides the details of the included studies. Among the 24 reports, 69.3% of the surveyed residents were female (*n* = 7448 out of 10,753 residents who indicated gender; range: 53.5% to 79.5%) [21,22,23,24,25,26,27,28,29,30,31,32,33,34,35,36,37,38,40,41,42,43,44,45].

Rekhi et al. [22] surveyed residents 60 years old or older. Rabiei et al. [24], Cornejo et al. [36] and Shivakumar et al. [23] surveyed residents 65 years old or older. The mean age of the surveyed residents in these investigations was not reported. Nineteen studies [21,25,26,27,29,30,31,32,33,34,35,37,39,40,41,42,43,44,45] reported the mean age of their surveyed cohorts, which was 72.2 to 85.5 years. Two studies [28,38] reported the mean age of their surveyed cohorts with reference to their gender: 79.1 to 85.7 years for females and 75.2 to 77.8 years for males.

#### 3.2.3. Systemic Conditions

Four studies [27,31,33,34] reported the body mass index (BMI) of their residents. Briefly, a Finish study [33] following 1369 residents reported that the mean BMI was approximately 25. Takeuchi et al. [27] dichotomized their residents into those with a BMI < 25 kg/m^2^ or those ≥ 25 kg/m^2^ and reported that 85.9% of the surveyed residents had a BMI < 25. An Italian study [34] reported that, of the 1326 surveyed residents, 9.2% were severely underweight, 15.1% were underweight, 27.8% were at a healthy weight, 34.2% were overweight and 13.7% were obese.

Four reports [21,29,35,36] indicated that residents with Mild Cognitive Impairment (MCI)/dementia were excluded from their studies. Eleven studies [24,25,27,30,31,33,34,38,39,41,42] included residents with cognitive impairment, comprising MCI and/or dementia. Except Takeuchi et al. [27], ten studies reported the prevalence of MCI/dementia among the surveyed institutionalized elderly population, which was 2844 of 5776 or 49.2% (range: 16.7% to 62.1%). Rather than the MCI/dementia state, Takeuchi et al. [27] reported the mean Mini-Mental State Examination (MMSE) score of the surveyed residents.

Other than the status of MCI/dementia, eight studies [24,25,28,29,30,32,38,44] reported the systemic conditions of the surveyed cohorts. Three studies [28,30,44], however, provided no details about individual disease, whereas one study [32] reported only the nature of the medications taken by the residents. Four studies [24,25,29,38] reported on the systemic disease prevalence (*n* = 1042) other than dementia (mean 29.6%, [0% to 38.0%])—cerebrovascular accidents (mean 16.2%, [0% to 21.8%]), hypertension (mean 16.1%, [0% to 34.2%]), diabetes (mean 14.3%, [0% to 24.7%]) and psychiatric disease/neuropsychological disorders (mean 7.0%, [0% to 37.7%]) were among the more common chronic illnesses. Zenthöfer et al. [30] used the pantomime test to assess apraxia (i.e. a disorder of higher motor cognition, considered a possible sequel of left hemispheric stroke) and reported that 57.6% of the 92 residents were affected.

The Charlson comorbidity index (CCI), which predicts the 10-year survival rate in patients with multiple comorbidities, was used in two studies [27,33]. Saarela et al. [33] reported that the mean CCI was approximately 2.9 for the 1369 Finish residents, whereas 69.2% of the 234 Japanese residents from the Japanese [27] survey had a higher CCI score, indicating a lower estimated 10-year survival rate. Additional information concerning the functional ability, nutritional status and oral health-related quality of life of the older institutional residents is available in Appendix 3.

### 3.3. Oral Health Status among Institutionalised Elderly Population 

The oral health of the institutionalized elderly population was evaluated based primarily on objective protocols and methods except for the prosthesis or denture status. Many studies reported unclear assessment protocols or guidelines for these. The evaluations were performed by oral healthcare providers, such as dentists, dental hygienists, dental/hygienist students and trained ward nurses (many reported examiners were calibrated), using the following validated protocols:

- Decayed Missing Filled Teeth (DMFT) Index (13 reports) [21,23,25,28,29,31,32,35,36,37,39,40,42],
- Root Caries Index (RCI) (1 report) [26],
- decayed root (2 reports) [29,37],
- Revised Oral Assessment Guide (ROAG) (1 report) [30],
- combined Plaque Index for Long-term Care (PI-LTC), Gingival Index for Long-Term Care (GI-LTC) and Denture Plaque Index (DPI) (1 report) [41],
- Visual Plaque Index (VPI) (2 reports) [26,38],
- Community Periodontal Index (CPI)/ Community Periodontal Index of Treatment Needs (CPITN) (8 reports) [23,29,30,35,36,38,41,46], and
- PSR including the Dutch version (2 reports) [31,32].

Details regarding dental attendance and supplementary oral health information are summarized in Appendix 3.

#### General Oral Problems Identified Through Oral Examination and Assessment

The included studies provided oral examinations to identify mucosal, dental, periodontal and temporomandibular joint (TMJ) problems, in particular, the prevalence and severity of those problems were accounted for among the institutionalized elderly population. Dental status was reported in 20 studies [21,22,23,24,26,27,28,29,30,31,32,33,36,37,38,39,42,43,44,45]. Based on the results of 13 studies [21,23,28,29,31,32,35,36,37,39,40,42,43] that reported the DMFT, the mean DMFT score ranged from 11.3 to 28.8 (missing teeth—15.0 to 24.9, decayed teeth—1.2 to 3.5, decayed roots—0.6 to 2.2 and filled teeth—0.2 to 8.0). Tan and Lo [26] used the RCI to evaluate the status of the exposed root surfaces and to characterize the root decay pattern. They reported a higher prevalence of decayed/filled lesions at the buccal, distal or mesial surfaces than the lingual surface in the residents. Decayed/filled lesions appeared more prevalent at the buccal surface.

Out of 22 studies [21,22,23,24,27,28,29,30,31,32,33,34,35,36,37,38,40,41,42,43,44,45], 4100 (42.7%) out of 9,606 surveyed individuals were reported to be edentulous, ranging from 20.4% to 62.0% edentulism among the institutionalized elderly population. Porter et al. [37] reported that more difficulties in eating were found in fully edentulous residents (*n* = 55) than in dentated/partially dentated UK residents (*n* = 124). Edentulous residents had more difficulty speaking, smiling, laughing or showing teeth without embarrassment (9.1%) and had more emotional problems (5.5%). Fully or partially dentated residents had difficulty cleaning their teeth or dentures (3.2%) and relaxing or sleeping (0.8%) but these problems were not found in edentulous residents. Dentated residents also had other oral problems, including broken teeth (23.9%), toothaches (17.1%), sensitive teeth (15.4%), loose teeth (15.4%) or bleeding gums (10.1%). Similarly, Kshetrimayum et al. [21] reported that fully or partially dentated Indian residents had more discomfort in eating and swallowing, more deterrents in speaking, more sensitivities in teeth and gums, more discomfort from eating in front of people and more limits in the variety and amount of food. Porter et al. and co-workers [37] reported that almost 70% of residents had un-rehabilitated anterior space.

For periodontal problems, the CPI protocol was applied in eight studies [22,23,29,30,35,36,38,42], whereas a conventional result presentation was given in only five studies [22,23,29,36,42]. The similar PSR protocol was applied in two studies [31,32]. The highest CPI score of the surveyed 2938 residents from the eight studies [22,23,29,30,35,36,38,42] ranged from 4.3% to 13.3% for deep pockets (4), from 8.3% to 31.7% for shallow pockets (3), from 18.6% to 38.3% for calculus (2), from 0.0% to 11.7% for bleeding on probing (1) and from 0.0% to 5.0% for healthy results (0), indicating a high prevalence of periodontal problems and the need for treatment. Using the PSR protocol, Ziebolz et al. [31] reported that 78.9% of the 46 surveyed residents had moderate or deep periodontal pockets. Janssens et al. [32] reported that, of the 143 Belgian surveyed residents, approximately 15% had the highest Dutch PSR score, indicating deep periodontal pockets (4), 53% had moderate pockets with complications (3*), 5% had moderate pockets (3), 24% had calculus (2) and 2% had bleeding on probing (1). Three studies [22,29,42] also reported the highest loss of attachment scores ranging from 0.0% to 11.7% for more than 12 mm (4), from 2.8% to 13.3% for 9 to 11 mm (3), from 9.9% to 30.0% for 6 to 8 mm (2), from 9.7% to 23.3% for 4 to 5 mm (1) or from 0% to 21.7% for 0 to 3 mm (0), indicating up to half of these elderly residents experienced significant (at least 9 mm) periodontal attachment loss.

Hopcraft et al. [38,58] reported that periodontal health was extremely poor among the surveyed subjects. Visible plaque was found in all residents and more than 25% of residents had plaque covering more than one-third of at least one index tooth. In addition, more than 50% of residents had calculus. They also found that female residents had increased visual plaque but male residents had increased levels of periodontal disease, such as periodontal pockets (> 4 mm). Janssens et al. [32,49] reported that, among the 143 surveyed Belgian residents, the mean Plaque Index (PI) was 2.1, indicating that oral hygiene was not ideal. Zimmerman et al. [41] reported slightly better plaque control (PI-LTC = 1.7 ± 0.8 (out of 3); DPI = 2.2 ± 1.2 (out of 4)) and gingival conditions (GI-LTC = 1.5 ± 0.9, (out of 4)) among a group of US residents in 11 long-term care homes where services from six dentists and two hygienists were available. The Canadian study by Kotzer et al. [40,60] reported a debris index ≥ 2 associated with ≥1 D crown based on a logistic regression analysis.

Regarding denture problems, 12 studies [23,28,30,32,33,34,35,36,40,42,43,45] reported some sort of denture or prosthetic status of the surveyed elderly. Among them, three studies [23,36,42] used the protocol from the World Health Organization (WHO) Oral Health Surveys [62] to assess the denture status, denture requirements and functional edentulism state and one study each used the assessment scale by Vervoorn et al. [40,60,63] or the denture-related subscales of ROAG [30] to characterize the denture status of their residents. The remaining eight reports, however, did not specify the denture or prosthesis status assessment criteria. Denture problems were found in both partially dentate and edentulous residents. Three reports [23,36,42] observed approximately 45% (upper) or 35% (lower) of surveyed residents used removable prostheses, whereas the corresponding prosthesis requirements were around 55% (upper) or 70% (lower) for removable prostheses. They reported that approximately 75% of the residents were considered functionally edentulous. Of the existing dentures, approximately 40% were judged to be unretentive, 30% were unstable or required rebasing and 10% needed repair.

Seven studies [28,30,32,33,41,43,45] reported the types of prosthesis used by residents. Of the 1702 edentulous participants (from total *n* = 3767), 1401 had complete dentures (82.5%, [47.8% to 100%], mean 86.5%), 246 had no replacement (14.5%, [0.0% to 23.6%], mean 9.5%) and 52 had complete upper dentures (3.1%). Three reports [28,43,45] stated that 60 out of 497 residents (12.1%, [1.9% to 25.4%], mean 16.6%) had their own natural dentition, needing no prosthesis, whereas five studies [28,30,41,43,45] indicated that 225 (19.2%, 13.8-28.6%, mean 21.5%) of the 1172 residents wore removable partial dentures. In general, denture hygiene was considered moderate or poor [28,31,41,56]. Mozafari et al. [25] reported the type and duration of denture wearing. Two studies [41,56] reported fairly poor denture plaque control (DPI = 2.2 ± 1.2 (out of 4)) and poor denture hygiene (DHI of 82.9) of the residents. A median of 21.5% (ranging from 10.5% to 54.6%) of the surveyed residents had some sort of denture stomatitis [24,25,28,29,40]. Other denture-associated complications included denture-induced hyperplasia and angular cheilitis [24,25,28,29,40]. Only Santucci and Attard’s studies [35,51] objectively investigated denture satisfaction of the Maltese residents using a validated denture satisfaction questionnaire [64]. Moreover, Porter et al. [37] reported British residents’ self-rated denture status.

Regarding chewing function and functional tooth units (FTUs), four studies [27,34,35,43] reported on functional dentition (categories: 0, 1–10, 11–20 or >20 [43]; or ≤20 vs. >20 [35]), FTUs, including tooth, fixed or removable prosthetic units [27] or functional dental units (FDUs) including natural, artificial (i.e., fixed pontics) or removable (i.e., denture units [34]) units among the surveyed institutionalized elderly population. Kim et al. [43] and Cocco et al. [34] reported that many of the surveyed residents had inadequate functional units for chewing. Santucci and Attard [35] reported that only 8% of the assessed residents had a functional dentition of ≥21 teeth.

The Japanese report focused on the relationship between FTUs and the functional dependency of the surveyed residents [27], whereas the Italian group identified whether an association exists between FDUs and the MMSE score of the surveyed participants [34]. For functionally independent Japanese residents, the mean FTUs was 10.7, whereas the corresponding mean FTUs for functionally dependent residents was 8.7. Cocco et al. [34] observed that over 86% of the surveyed residents had insufficient FDUs. No detail was given regarding how good, sufficient or insufficient FDUs were defined.

Regarding oral mucosal problems, four included studies [22,24,25,37] reported poor oral mucosal conditions in older institution residents. Porter et al. [37] reported 41% of their surveyed UK residents had dry mouth and about 35% had dry, sore or cracked lips. Mozafari et al. [25] reported that 98% of northern Iranian residents had at least one prevalent oral mucosal lesion. Other issues included atrophic glossitis (48.5%), dry mouth (38.1%), atrophic tongue (38%) and burning mouth (16.7%). Another two studies investigating north-western Iranian residents [31] or Indian residents living in Delhi [29] also reported similar findings and even more severe oral mucosal conditions, including erythematous and pseudomembranous candidiasis, traumatic ulcer, leukoplakia, erythroplakia or lichen planus.

Some studies also used integrated oral health measurements using the ROAG. For example, Zenthöfer et al. [53,57] reported that more than 50% of residents were identified to have poor oral health (mean ROAG score 2.3) in southwest German nursing homes.

### 3.4. Oral Health-Related Quality of Life among Institutionalised Elderly 

The OHRQoL was evaluated using the GOHAI (8 studies) [21,22,23,30,35,36,42,45], OHIP-14 (3 studies) [35,40,43] or OIDP (one study) [37] in 11 of the 25 included studies/study series. Due to the variations in result reporting and data analysis and the limited number of studies included using the same OHRQoL or oral health assessment tools, a descriptive report was conducted. The eight studies [21,22,23,30,35,36,42,45] using the GOHAI reported that the overall score ranged from 32.9 to 51.6 out of 60.

Regarding the three studies that used the OHIP-14 tool [35,40,43], the overall OHRQoL results from the groups appeared to be rather different. Kim et al. [43] reported the overall mean of the OHIP-14 of the South Korean older institutional residents was 10.3. Kotzer et al. [40] compared the OHIP-14 between older Canadian community and institutional residents. They reported that the seniors from long-term institutions (*n* = 297; mean 5.71) appeared to score more poorly on the OHIP-14 than the community group (*n* = 501; mean 4.75). Santucci and Attard [35], however, reported a lower or apparently better OHIP-14 score of 3.8 for Maltese residents.

### 3.5. Factors Associated with Oral Health among Institutionalised Elderly

Factors associated with oral health could be conceptually categorized as either non-modifiable or modifiable factors. Non-modifiable factors include age and gender, acquired systemic health conditions (MCI/dementia, medication use, etc.) and modifiable factors consist of oral health conditions, general health conditions (e.g., nutritional status) and others. Due to the diversity of reporting styles, data analysis protocols and the limited number of studies reporting the same or similar factors or parameters associated with the oral health of the institutionalized elderly population, a descriptive report was conducted. 

#### 3.5.1. Non-Modifiable Factors

Regarding the factor of age, 14 studies [22,23,24,26,27,28,29,30,32,33,34,38,39,45] investigated whether age was associated with various degrees of poor oral conditions and a poor OHRQoL. Four studies/study series [29,32,33,34] reported that age is associated with dental problems. Through a bivariate analysis, Özkan et al. [29] reported that 75- to 84-year-old Turkish residents had more decayed teeth or roots and filled teeth. Cocco et al. [34] performed a multinomial logistic regression analysis and reported that, in Italians, ≥80 years of age was associated with reduced FDUs. The study series by Janssens et al. [32,49] reported that older dentate residents had a higher proportion of decayed teeth according to the mixed-effect logistic regression analysis. Saarela et al. [33,50] reported that dentition status was associated with age but no further analysis was presented for the association between dentition status and various age ranges.

Kotzer et al. [40,60] reported that the age range of 65 years or older in residents was associated with a debris index ≥2 based on a logistic regression analysis. In addition, Janssens et al. [32,49] applied a general linear logistic mixed analysis of their data and identified that older Belgian residents had higher odds of full denture wearing.

Two study series reported on periodontal problems for various ages. A Brazilian study series [42,61,65] indicated that the prevalence of periodontitis was 20.6% in residents older than 64 years, whereas the prevalence was 45.7% for residents who were 75 to 84 years old. A lower prevalence of 14.3% was observed for residents who were 95 years old or older. Hopcraft et al. [38,58] performed a logistic regression data analysis and reported that Australians from 75 to 84 years old were associated with a CPI score of 3 or above (i.e. shallow (4 to 6 mm; in 35.6% residents) or deep periodontal pocket (>6 mm; in 10.2% residents) in any one sextant of a resident. The residents who were 75 to 84 years old also had increased visual plaque.

Three studies [22,23,45,46,52] reported that age was associated with a poorer OHRQoL measured by the GOHAI based on a bivariate analysis. Moreover, the German study series [30,56] reported a significant association between age and tooth loss, whereas lower odds of a reduction in the OHRQoL were found in older residents. Upon a multivariate logistic regression analysis, a Brazilian study indicated that older age was significantly associated with a compromised GOHAI [42]. Kotzer et al. [40], using logistic regression, found that Canadian urban home residents who were 75 years old or older were associated with complete upper and lower dentures fairly/very often according to OHIP-14.

Regarding gender, 11 studies [21,22,24,30,32,33,34,36,38,42,45] explored the association between gender and oral problems or a poor OHRQoL. Three study series [32,38,42,49,58,61,65] reported that the male gender was associated with dental/periodontal problems. Janssens et al. [32,49] reported that male Belgian dentate residents had a higher proportion of decayed teeth according to a mixed-effect logistic regression analysis. Hopcraft et al. [38,58] indicated that Australian male residents were associated with a CPI score of 3 or above, indicating possibly poor periodontal conditions detectable by logistic regression analysis. A Brazilian study [42,61,65] used an adjusted multivariate data analysis and reported that males appeared to be associated with higher rehabilitation needs.

Alternatively, more studies [30,36,53,55,56,57] reported a significant association between the female gender and oral problems or the OHRQoL. Cornejo et al. [36] reported that Spanish female residents had poor dental and periodontal conditions, with a high prevalence of calculus, 4 to 5 mm pockets and a higher prevalence of edentulism than males. Interestingly, Janssens et al. [32,49] also reported that dentate female residents had higher treatment needs, whereas younger female dentate residents were associated with filled teeth. In contrast, the German study series by Zenthöfer et al. [30,53,54,55,56,57] showed a significant association between poor CPITN scores (reported unconventionally) but not with GBI or DHI and female residents. Saarela et al. [33,50] reported that the dentition status of Finish residents was associated with gender, but the corresponding data or details were not provided to substantiate these claims. Cocco et al. [34] reported that female residents had significantly reduced FDUs. Rabiei et al. [31] indicated that female Iranian denture wearers were at a higher risk of denture complications, such as denture stomatitis and overall, these Iranian institutionalized females had a significantly higher prevalence of oral mucosal disorders. A Brazilian study series [42,61,65] reported that females were associated with more TMJ alterations.

Niesten et al. [45,52] conducted an adjusted multivariate analysis and reported that Dutch female residents were associated with low GOHAI scores. Rekhi et al. [22,46] performed a bivariate analysis and reported that Indian female residents also had a poorer OHRQoL measured by the GOHAI. Moreover, Kshetrimayum et al. [21] performed a multiple logistic regression data analysis and reported that the Indian female institutionalized elderly population had poor nutritional status.

In terms of the educational level, four studies reported the association between the education level and the poor oral health of the institutionalized elderly population [22,28,33,40]. Rekhi et al. [22] reported that Indian residents without formal education were perceived to have a poorer OHRQoL. Kotzer et al. [40] conducted a logistic regression data analysis and reported that Canadian residents with a high school education or less were 2.3 times more likely to report an OHRQoL effect ‘fairly often’ or ‘very often.’ Saarela et al. [33,50] reported that Finish residents’ dentition status was associated with education, but no further analysis was attempted to clarify the relationship.

Regarding the acquired systemic conditions, among eight studies [25,28,29,30,32,38,41,44] that reported on the systemic conditions of the residents, only one German report [30] investigated how the systemic conditions might be associated with poor oral health or an inferior OHRQoL. In addition, five studies [21,27,31,33,34] reported on the nutrition status of the older residents. Saarela et al. [33,50] reported that Finish residents who were edentulous and without dentures predicted low protein intake (<60 g/day) based on an adjusted logistic regression analysis. However, using a multivariate analysis, only one study [31] reported that edentulism might be associated with malnutrition in older residents.

In assessing dementia or other cognitive impairments, 10 studies [24,25,27,30,31,33,34,38,39,41] included residents with such impairments, including MCI and dementia. Five studies [30,31,33,39,41] reported that dementia was significantly associated with poor nutrition, oral health or OHRQoL. Moreover, Ziebolz et al. [31] performed a multivariate analysis and reported that north-western German institutionalized elderly with dementia had a higher risk of malnutrition.

Zimmerman et al. [41] performed a bivariate analysis and reported that dementia was associated with the PI-LTC in surveyed US residents. Philip et al. [39,59] reported that dementia was associated with a higher mean score for full mouth O’Leary’s plaque and increased gingival inflammation in Australian institutionalized elderly. Zenthöfer et al. [30] reported on a multivariate logistic regression data analysis that indicated that dementia was significantly associated with an unconventionally presented CPITN score.

Takeuchi et al. [27,47] reported that total FTUs were positively associated with the MMSE scores in Japanese residents after adjustment for age, sex and the number of natural teeth based on the multivariate analysis results. Similarly, via multinomial logistic regression data analysis, Cocco et al. [34] found that low MMSE scores for Italian residents were associated with reduced FDUs. In addition, Zenthöfer et al. [30] used the Spearman correlation and reported that dementia was associated with a poor OHRQoL in south-western German residents.

#### 3.5.2. Modifiable Factors

In terms of dental attendance and service accessibility, seven studies [23,28,29,38,40,41,42] reported on the frequency of dental visits of the surveyed institutionalized elderly population. Among these studies, approximately 20% to 75.3% of residents had a dental visit within the past 12 months. Six of 14 (42.9%) nursing homes from a US study [41] had dental services provided for the residents. A Turkish study [28] reported that 38% of the surveyed residents had had dental visits within one year driven by oral symptoms. A later report [29] from the same country indicated that only 4.4% of residents visited a dentist regularly and about 70% indicated irregular attendance. Hopcraft et al. [38,58] reported an association between dental visits and oral health problems. They also found that assistance with tooth brushing and the frequency of tooth brushing were significantly associated with better oral hygiene. Piuvezam et al. [42,61,65] showed that Brazilian residents whose last dental visit was longer than 1 year past were significantly associated with higher rehabilitation needs based on an adjusted multivariate analysis.

Five studies/series [21,36,40,42,43,60,61,65] reported an association between perceived dental treatment needs and oral health problems or the OHRQoL. Piuvezam et al. [42,61,65] reported that residents who perceived that their gums, teeth or prostheses were fair required more extractions. A Canadian study series [40,60] reported that residents’ perceived oral/dental treatment needs were associated with ≥1 decayed/filled roots based on a logistic regression analysis.

Based on a bivariate analysis, Kim et al. [43] reported that poor oral health status, poor self-reported oral health and concern regarding oral health among South Korean residents were associated with a worse OHRQoL as measured using the OHIP-14. Rekhi et al. [22,46] reported that poor perceived general/oral health or perceived dental treatment needs were significantly associated with poorer GOHAI scores in Indian institutionalized elderly. Cornejo et al. [36] reported that Spanish residents who perceived teeth, gums or denture problems had a poor GOHAI. Similarly, the Canadian report series by Kotzer et al. [40,60] indicated that those surveyed residents with fair or poor perceived mouth health were associated with OHRQoL effects ‘fairly often’ or ‘very often.’

In terms of oral health-related factors, six studies [26,28,37,38,39,40] reported on how various oral-related conditions are associated with oral health or the OHRQoL. Oral hygiene by tooth brushing or denture brushing was reported in three studies [28,37,40]. A Canadian study series [40,60] reported that tooth brushing less than one time per day was associated with a debris index of ≥2. A Turkish study series [28,48] reported that poor denture hygiene habits are significantly associated with stomatitis. Tan and Lo [26] applied a multilevel logistic regression data analysis and showed that root surfaces with denture contact and increased gingival recession were associated with increased decayed/filled lesions, indicating one possible consequence of difficult or inadequate denture hygiene.

Regarding dental or gum-related factors, nine studies [21,22,30,31,35,36,37,38,45] characterized interactions between various dental factors and poor oral health. Using a logistic regression analysis, two reports [31,38] investigated various dental-related conditions associated with oral health problems. Hopcraft et al. [38,58] reported that having more than nine natural teeth or poor plaque control was associated with a CPI score of 3 or higher, indicating moderate to advanced periodontitis in Australian residents, whereas Ziebolz et al. [31] reported that German edentulous residents were associated with a risk of malnutrition.

The remaining seven studies [21,22,30,35,36,37,45] reported the association between dental-related conditions and the OHRQoL. Santucci and Attard’s study series [35,55] performed Spearman’s correlation and reported that Maltese residents with increased decayed teeth, missing teeth or DMFT, without maxillary/mandibular dentures were associated with a better OHRQoL (OHIP-14/GOHAI-20). Zenthöfer et al. [30] performed a multivariate logistic regression and reported that a compromised GOHAI is significantly associated with the absence of prosthesis rehabilitation and fewer than five standing teeth. Porter et al. [37] performed an adjusted multivariate logistic regression and reported that the prevalence of oral effects in UK residents (i.e., at least one OIDP item with a non-zero score) was associated with sensitive teeth, toothache and loose natural teeth.

Cornejo et al. [36] and Shivakumar et al. [23] performed a bivariate analysis and reported that residents who were functionally edentulous were associated with a poor GOHAI score and those who needed upper or lower prosthesis were associated with a poor perception of oral health. Shivakumar et al. [23] also reported that unmet dental care needs were associated with a poor OHRQoL measured by the GOHAI. Niesten et al. [45,52] performed a one-way analysis of variance (ANOVA) and reported that a higher/better GOHAI score from Dutch residents was found to be associated with the absence of caries, no clinical treatment needs and no reported treatment demand. Rekhi et al. [22,46] reported that, upon a bivariate analysis, excluding CPI sextants, having decayed teeth or needing various dental prostheses among Indian residents was significantly associated with a poorer OHRQoL measured by the GOHAI. However, no multiple logistic regression analysis was performed in these studies.

Concerning denture-related factors, seven studies [25,28,30,35,36,42,45] reported on whether denture-related conditions are associated with oral health or the OHRQoL. Uludamar et al. [28] reported that a higher proportion of Turkish residents who did not have proper dental or denture care experienced denture stomatitis, which is associated with age, income, general health, denture hygiene and overnight denture wearing based on distribution. Mozafari et al. [25] claimed that the duration of denture wearing in Iranian residents was associated with denture-related or oral mucosal lesions. The Brazilian group [42,61,65] reported that residents needing upper dentures were associated with more TMJ alterations. The Dutch study series [45,52] indicated that edentulism was associated with the frequency or change in dental service use. However, no multiple logistic regression analysis was performed in these studies.

Santucci and Attard’s study series [35,55] performed Spearman’s correlation and reported that Maltese residents’ denture satisfaction was associated with denture age and denture type. Similarly, Zenthöfer et al. [30] reported that the type of denture appeared to be associated with the OHRQoL (fixed partial dentures, removable partial prostheses or complete dentures). The authors performed a multivariate logistic regression and reported that the compromised GOHAI was significantly associated with wearing dentures and insufficient denture conditions. Approximately 2.5-fold odds of a poorer OHRQoL were found in German residents with denture-related treatment needs. Cornejo et al. [36] performed a bivariate analysis and reported that Spanish residents who needed upper dentures were associated with a poor GOHAI. Kshetrimayum et al. [21] reported that Indian dentate residents had a significantly better OHRQoL than edentulous residents (mean GOHAI of 49.0 vs. 41.2).

In terms of self-care independence, six studies [27,30,32,38,39,45] reported that self-care dependence was significantly associated with poor oral health or OHRQoL. Niesten et al. [45,52] reported that Dutch residents who had high care dependency or could not brush had a lower teeth-brushing frequency. In effect, 29.4% and 13.5% of Dutch residents with medium to high care dependency or limited dental brushing had poor dental and oral hygiene.

Hopcraft et al. [38,58] reported that fewer than one-third of residents clean their teeth more than two times a day and that 50% of residents reported cleaning their teeth only once a day. The authors reported that residents who require more assistance with oral hygiene had more decayed teeth and fewer filled teeth. A separate study by Philip et al. [39] reported that partially disabled or disabled Western Australian institutionalized elderly needing assistance with oral care were associated with a higher mean of the O’Leary’s plaque score and gingival inflammation.

Takeuchi et al. [27,47] reported that Japanese dependent residents had fewer FTUs. Upon logistic regression analyses, functional dependence was associated with posterior teeth occlusion. More FTUs were associated with greater odds of independence for essential personal care, whereas the loss of posterior teeth occlusion was independently associated with cognitive decline. The authors also reported that the total FTUs were positively associated with MMSE scores after adjusting for age, sex and number of natural teeth based on the multivariate analysis results. The authors also reported that more FTUs were significantly associated with greater odds of self-care independence.

Janssens et al. [32,49] reported that medium care dependency, increasing age and possession of a preferential tariff among surveyed Belgian residents were significantly associated with higher odds of wearing a full set of dentures based on the results of a general linear logistic mixed analysis. In addition, a German study series [30,53,54,55,56,57] reported that, using the Spearman correlation, the ROAG was found to be associated with dependency (Barthel Index) and dementia (MMSE). They also reported that dependency was associated with the OHRQoL.

Regarding medications and hyposalivation, Zenthöfer et al. [56] reported that residents taking coagulation inhibitors were significantly associated with a 2.2-fold risk of poor oral health, particularly in periodontal conditions (CPITN, reported unconventionally). Upon a multivariate logistic regression, Zenthöfer et al. [55] reported that their unconventionally presented CPITN data were significantly associated with female gender, dementia and coagulation inhibitor use. Caution is recommended in interpreting the related data because of their unusual usage of the CPITN data format.

Four studies [24,25,33,37] investigated whether the number of medications caused hyposalivation or dry mouth but only one [37] reported that dry mouth was associated with poor oral health. Brukiené et al. [44] investigated the salivary flow rate, pH and buffer capacity and found that a slow salivary rate and lower pH were reported by Lithuanian residents. The authors also reported a negative correlation between the salivary flow rate and number of systemic diseases based on a bivariate analysis. Brukiené et al. [44] reported a negative correlation between Lithuanian residents’ salivary flow rate and the number of systemic diseases. The same analysis also reported a negative correlation between the number of standing teeth and number of medications used. The Pearson correlation showed that the salivary buffer capacity was significantly associated with the salivary pH.

Janssens et al. [32] reported the potential hyposalivary effects on Belgian residents with prescribed medications. Upon a bivariate analysis, the number of medications (>10) potentially induced dry mouth, particularly in residents who are dentate with more natural teeth and increased treatment needs. Moreover, Porter et al. [37] performed an adjusted multivariate logistic regression and reported that the prevalence of oral effects (i.e., at least one OIDP item with a non-zero score) was associated with bleeding gums and dry mouth among the UK institutionalized elderly population.

Only a Canadian study series by Kotzer [40,60] reported that smoking years were associated with ≥1 decayed/filled roots and DMFT in dentate residents based on a logistic regression analysis. In terms of other geographic or socioeconomic factors, five studies [32,41,42,43,45] explored the association between various geographic/socioeconomic factors and poor oral health or the OHRQoL.

Zimmerman et al. [41] performed a bivariate analysis and reported that, in profit-making US nursing homes, a lower proportion of residents under Medicaid, a dental hygienist visit in the last quarter, a shorter length of stay and currently being on hospice were associated with lower PI-LTC. Poorer denture hygiene was also found to be significantly associated with private pay status. Janssens et al. [32,49] reported that the possession of a preferential tariff among Belgian residents was significantly associated with higher odds of full denture wearing upon a general linear logistic mixed analysis. They also found that having no preferential tariff was associated with filled teeth or the restorative index. The Brazilian study series [42,61,65] conducted an adjusted multivariate analysis and reported that lacking private health assistance or not being located in a southern Brazil region was associated with higher rehabilitation needs. The accessibility to health services and type of institution were significantly associated with the OHRQoL, whereas geographic regions were associated with oral health. The authors found that the residents with missing teeth from the south and south-east Brazil had significant rehabilitation and extraction needs.

Kim et al. [43] performed a bivariate analysis and reported that the inability to pay a dental bill and a low number of chewable food items were associated with a worse OHRQoL measured by the OHIP in South Korean residents. In a Dutch study series [45,52], difficulty in visiting the dentist or the existence of clinical treatment needs were associated with the frequency or change in dental service use. However, no further logistic regression analyses were attempted. 

## 4. Discussion

Twenty-two of the included studies used a cross-sectional design, whereas three adopted a case-control design. The methodological quality of most of the studies ranged from moderate to strong (Table 1 and Table 2). The reasons for these studies not achieving a higher quality standard were often because of the following:

- The time for the research objects was not well defined;
- Convenience sampling or a lack of sampling strategy/randomization was used;
- No proper sample size estimation was calculated, particularly in reference to the nature of the dependent variable and number of independent variables to be analyzed and the subsequent regression analyses, including the control protocol for the confounders.

The methodological quality of these studies may also affect the quality of this review. Because these studies were cross-sectional and examined the outcome measures at one point in time, the lack of a longitudinal design, randomization, and/or comparison methods may affect the validity and consistency of the results on oral health problems and the factors associated with oral health among institutionalized residents.

In general, the included 25 studies/series exhibited no standardized reporting style and some did not include clear a summary of the primary data or essential background information of the surveyed residents. As a result, only a small proportion of studies reported the dependency level, malnutrition risks, MCI/dementia proportion or systemic conditions. Concerning oral health and OHRQoL data, the situation appeared slightly better; however, the varied recording protocol and disease categorization criteria also limited the generalizability of the observations across different reports.

This review investigated the relationship between the aspects of oral health and the associated factors on oral health problems and the OHRQoL among the institutionalized elderly population. The results confirm that this population comprises vulnerable people who have poor oral health because they are relatively weak and dependent [66]. The identified non-modifiable and modifiable factors associated with vulnerable oral health among this underprivileged group could be categorized as individual or environmental factors.

Individual factors are most likely due to limited physical functioning and/or cognitive impairment, leading to a limited self-care ability [30,31,37,38,41,56]. Although the limited data could be extracted and therefore reported in this review, non-communicable diseases are common in elderly, institutionalized or not [67]. The current report suggests the non-communicable diseases likely to increase physical inability and self-care dependence [30,32,37,38,45]. Based on the evidence-based findings of the relevant included studies over the past decade, the institutionalized elderly population is at increased odds to have poor oral health with various degrees of dental, periodontal, oral mucosal and TMJ problems and poor functions due to missing teeth, inadequate replacement or poor dentures. Oral problems cause oral distress, poor function and a deprived OHRQoL. Chronic oral distress and poor chewing function could affect general health, such as through the increased risk of malnourishment. The outcomes of this review are comparable to those of the previous findings [68,69]. Notably, maintaining oral hygiene is a decisive factor in good oral and general health [58].

The oral examinations were performed by dentists, dental hygienists, dental/hygienist students, trained ward nurses or calibrated examiners using varied protocols, with some not accounted for in detail. With that, the outcomes of the oral health evaluations were diverse and, oftentimes, non-generalizable and hard to compare. Nevertheless, this review found that poor oral hygiene in residents was probably due to inadequate teeth/denture brushing or cleaning, which increases the prevalence of dental problems and periodontal diseases or other severe complications. In addition, in this special group, the related predisposing factors were considered to be MCI/dementia, high care dependency and lack of access (except in a few North American institutions) to dental and oral care. The most common dental problems included large amounts of plaque, debris and calculus and moderate to advanced periodontitis. Periodontitis is a common oral problem in middle age or elderly populations, particularly in institutional residents [30,70]. All residents were found to have plaque and more than 70% of residents had plaque covering one index tooth. Poor oral hygiene indicates a higher possibility of gingivitis, periodontitis, periodontal attachment loss [38,41] and tooth loss [37]. In the elderly population suffering from hyposalivation due to ageing or concurrent medications, caries of crown and roots, tooth loss and so on are common [37]. Dental problems also increase the risk of oral health complications and general health problems [21,57,70,71].

As reported in the population or other special groups, good dental or oral health conditions are positively associated with the OHRQoL in the institutionalized elderly population. The number of natural teeth or FTUs/FDUs and, hence, the presence of proper, well-maintained oral rehabilitation are important in maintaining oral function, oral health and the OHRQoL [42,57,72]. This is because they facilitate masticatory performance that promotes chewing, enables a variety of food selection and processing and promotes the enjoyment of eating and, ultimately, a balanced diet. Such oral function is important in maintaining physio-psychosocial well-being [73]. Physically, adequate teeth or full denture units/full tooth units (FDUs/FTUs) maintain masticatory performance, which is important for satisfactory nutrition intake, normal social interactions and enhanced psychosocial satisfaction [73,74]. However, when dentate residents have untreated dental problems, they can experience discomfort while eating, increased sensitivity to extreme temperatures in foods and chronic or acute pain or ulcers in the mouth, ultimately causing difficulty in eating, limited choice of food, and, sometimes, difficulty speaking. Subsequently, they experience more psychosocial concerns, such as embarrassment and discomfort, when they eat in front of others. As a result, they take in a limited amount of food [37], leading to nutritional problems as well.

The evidence that this report reviewed so far has indicated that oral health is of great concern, particularly for the institutionalized population. Better oral hygiene increases the number of retained teeth and improves the quality of these teeth [61]. Strategies for the prevention of tooth loss and for maintaining adequate oral hygiene and preventing oral diseases at an early stage for an optimal OHRQoL among institutionalized residents are key for increasing the oral health of residents. Our review found that even a basic twice-a-day tooth-brushing habit seemed difficult to achieve, as more than 70% of the institutionalized residents undertook tooth brushing or cleaning only once per day or even less [58] and they did not have regular yearly dental visits [36,58]. More than 90% of the residents did not attend a dental visit more than once per year. The elderly population typically visits a dentist only in a symptom-driven mode (e.g., due to pain or discomfort), rather than attending regular recalls/check-ups for disease prevention and health maintenance [75]. Uncomfortable or painful treatments may lead to the refusal of future dental consultations and worsening oral health status [42,75,76].

The results of this review showed that a high percentage of residents were fully edentulous (approximately 42%) or were functionally partially or fully edentulous (<20 remaining teeth; approximately 75%). Most residents (>65%) needed upper or lower dentures [41,42,57], whereas a fair proportion of the surveyed denture needs were unmet (at least an estimated 20%). Wearing dentures, including complete or partial dentures, can help improve physical functions, such as chewing, communication, speaking and smiling [14,37]. Types of dentures and the fitness of dentures significantly affect the OHRQoL. Dentures that do not fit can reduce chewing efficacy, leading to an impaired OHRQoL [21,37,57,71]. However, the dentures require adequate daily oral hygiene and aftercare to maintain good oral function and health [37,41,57]. Therefore, it is crucial that maintaining oral hygiene is a top priority, which will result in better oral health.

Our review showed that both male and female residents had dental and/or periodontal problems [36,38] and structural TMJ alterations [42,61,65]. Although female residents were found to have poorer oral health and OHRQoL, inadequate evidence exists to support this finding at a statistically significant level. Regarding the effect of age on oral health, the results showed that increasing age generally increases the risk of a poorer OHRQoL among institutionalized elderly [57]. The oral hygiene and periodontal conditions of institutional residents aged 75 to 84 years old were more concerning, probably due to increased self-care dependence attributed to cognitive disability and multiple systemic chronic diseases. In this review, older residents were found to require more assistance, especially those who have cognitive disabilities, such as MCI/dementia and those who are unable to perform regular and proper daily oral care [36,57].

In general, approximately 20% to 50% of elderly people aged 65 or older have lost all of their natural teeth and around 15% were without complete dentures. For those who had complete dentures, many were ill-fitting or needed repair. Together those affected have multiple functional problems and an impaired OHRQoL. An estimated 10% of the institutionalized elderly population had natural functional dentition and the rest (est. 50%) were partially dentate and required prosthetic rehabilitation. Older residents who have inadequate natural teeth were also susceptible to poor oral health, such as periodontitis, tooth crown or root caries and so on. Poor oral or dental health in the institutionalized elderly population causes oral pain and discomfort, eating problems, weight loss, speech difficulties, nutritional problems (such as risk of malnutrition), predisposition to multiple non-communicable diseases (such as stroke or aspiration pneumonia), poor immunity, poor diabetic control [77], psychosocial distress and restriction [21,37]. In fact, the association between various systemic conditions and poor oral health was well recognized. Among the top five systemic diseases/conditions of the older institutional residents extracted from three of the 25 reviewed reports [30,32,44], the associations were also readily observable among dementia/MCI residents for incidence, risk of dementia, depression/anxiety and teeth lost [27,34,78,79], which perhaps is related to poor self-care and oral hygiene [39,41,52]. Regarding the remaining four systemic conditions, the two-way relationship between diabetes mellitus and poor periodontal health [80] was among the most well established. Along such lines, Hopcraft et al. [38,58] reported that dentated Diabetes Mellitus (DM) residents had more missing teeth. For the remaining three diseases, associations between atherosclerosis, cerebral vascular accidents and poor oral/periodontal health [81,82,83] were reported, probably related to poor oral care and, perhaps, to the direct or indirect effects of periodontopathic bacteria [84]. Meanwhile, hypertension and increased subgingival colonization of periodontal bacteria and, hence, periodontitis risk [85] were considered mechanisms underpinning the corresponding poor general health. Due to the heterogeneity of data collection, presentation and analysis protocols, no correlation between the latter three diseases/conditions and oral health was reported or observable (Table S6).

Oral health affects physical and psychosocial well-being and can lead to malnutrition, a serious physical problem that is more prevalent in residents with poor oral health [86,87] and a poorer OHRQoL [86,88]. The findings of this review emphasize the positive association between nutritional status and oral health or the OHRQoL.

The effect of the educational level on the OHRQoL was reported in four studies [22,28,33,40] in this review. Residents with a lower educational level had a poorer OHRQoL or poor dental health but the result was inconclusive because each study reported the respective result without analyzing the interactions between educational levels on oral health or the OHRQoL. Other studies [89,90] on oral health showed similar results, where an individual with a higher educational level perceives better oral health or OHRQoL because the individual may have more knowledge about oral problems and the related preventive measures, such as interdental cleaning. Better education develops positive attitudes and behaviors towards oral health [89,90]. Further study in this area for the better oral health of nursing home residents is warranted.

Furthermore, older residents have more medical health conditions with comorbidities and multiple medications are needed. Medications have side effects and often one of these is dry mouth. This review found a negative association between medication use or the number of medications used and oral health or the OHRQoL. Studies have shown that xerostomia or hyposalivation is a major risk for oral health [91,92,93,94]. However, of the three reviewed studies [25,32,44,49], only two [25,44] reported the prevalence of xerostomia. One study [44] investigated the salivary flow rate and the pH of saliva. However, detailed information was not given or analyses were not performed in this study. No study correlated the salivary flow rate or signs of dry mouth against the presence or absence of systemic diseases and the related quality or quantity of medications taken. The question regarding any association between medication use and poor oral health in the institutionalized elderly population has thus remained unanswered and warrants further investigation.

Two German studies in this review reported that residents with dementia appeared to have more standing teeth; however, their oral hygiene and oral health were compromised [30,31]. Residents who experienced dementia or cognitive impairment had poorer oral health [69,89,95,96,97]. Dementia, as one of the associated factors, reduces residents’ self-care ability and causes poorer oral hygiene, as reported in various studies [4,41,56,95]. This review reconfirms the observation that most residents with dementia appeared unable to perform adequate and regular oral care because they often forget about it [90,95,98]. They typically underwent tooth brushing once per week or less. The patient population with dementia requires double the rate of oral care assistance compared to those without dementia [38,57,95]. The oral health maintenance of the institutionalized elderly population with declined cognitive conditions is indeed a recognized burden on dental and healthcare systems [69,89].

Environmental factors mainly include oral care-related support from the community or society for the institutionalized elderly population, including service accessibility and financial or insurance support. As most of the institutional residents are dependent for self-care due to their physical disability, institutional caregivers perhaps need to play an important role in maintaining oral hygiene and promoting the oral health of this population. Recent studies have reported that oral hygiene and the health of the institutionalized elderly population, particularly in those with cognitive problems, are usually poor, which is attributed to a disregard for the need for oral healthcare among them and may also be due to the inadequate knowledge and skills, poor attitudes and improper practices of caregivers [96,97,99,100,101]. Oral care is usually not treated as a priority or a routine, resulting in delayed oral assessment and treatment [37,101,102]. The reasons for inadequate oral care practices among caregivers have been examined and were related to the following:

- time constraints and priority setting,
- insufficient education about oral care and skills,
- a lack of awareness regarding the importance of oral care in oral/dental diseases [103],
- an unwillingness to provide oral care due to the fear of managing uncooperative residents,
- the prevention of potential injuries, and
- avoiding an unpleasant task [104,105,106].

Therefore, it is important to train caregivers regarding appropriate oral care knowledge, skills and application [107].

Effective oral care to control plaque and promote oral healthcare includes proper tooth brushing and cleaning [37,38,41,98] and annual dental check-ups [42,75,76]. Primary care to promote oral health is crucial to help the institutionalized elderly population meet an acceptable level of oral health [37] and increase their awareness of oral healthcare [90,108]. To improve the motivation to regularly visit the dentist, an educational program should be developed for both institutionalized residents and caregivers. An educational program should aim to increase the awareness of oral care and hygiene to improve oral care practices. Apart from fostering an individualized understanding of oral care, regular dental check-ups enhance the outcomes. It is important to consider the accessibility of a dental clinic to the residents. The WHO encourages the development of cost-effective public policies based on a common risk-factor approach. Screenings for oral health problems should be performed regularly and more frequently, especially for at-risk residents, such as those with cognitive problems and dry mouth [89]. Considering the physical restrictions of frail elderly residents who are more dependent, a policy of frequent and regular on-site dentist visits to nursing homes is desirable as part of a dental healthcare scheme for the institutionalized elderly population. The policy should provide for oral checks, adequate oral care, removal of pain and immediate stabilization of any dental emergencies [31,38,57]. A specialized multidisciplinary approach is highly recommended to provide adequate and effective diagnoses and oral and dental treatments for institutional residents [42].

### Strengths and Limitations

This systematic review followed a rigorous systematic review methodology and adopted a comprehensive search strategy that included PICO and PRISMA and used various databases and hand-searching. To ensure the inclusion of updated and evidence-based knowledge, the literature search strictly followed the selection criteria and was limited to the past ten years. Two independent reviewers (F.M.F.W and Y.T.Y.N) and a third reviewer (WKL) ensured the eligibility of the included studies and the reliability of the data extraction and analyses. This review retrieved relevant studies/study series so that essential data were included to enhance the data analysis and interpretation. This was considered an important approach for a comprehensive search, data extraction and better understanding of these studies. The assessment of the methodological quality and bias of these studies was performed using the available objective tools. As almost all these studies were of a cross-sectional design, using the same quality checklist or assessment tool maintained the consistency of the evaluation. To ensure a thorough understanding of oral health among the institutionalized elderly population, both subjective and objective information related to oral health and the OHRQoL was collected from these studies. All information was extracted, analyzed and categorized into specific headings or subheadings to enhance the understanding of the constructs. Due to the diversity in reporting the outcome measures among the included studies, it was difficult to perform a meta-analysis.

Studies such as longitudinal follow-up investigations and double-blinded randomized controlled clinical trials (RCT) produce a higher level of evidence and enhance the overall understanding of changes in the oral health of older institutional residents and their OHRQoL. Such reports remain scarce. The results of this review cannot provide information about changes in oral health and the OHRQoL among the institutionalized elderly population. Moreover, the small number of included studies assessing a certain factor may reduce the general applicability of the review findings. Few studies on this topic with various focuses from North American (2), South American (1) or African countries (0) could meet the selection criteria, leading to a limited transferability of the review results to the corresponding institutionalized populations. 

## 5. Conclusions

This systematic review provides aggregated information about oral health and its associated factors among elderly institutional residents. Oral health was determined by oral examination with or without assessment tools and the OHRQoL was evaluated. Multiple oral problems with various levels of severity were observed. Factors associated with poor oral health were identified and they imposed complex and multifaceted influences on oral health. This review expands the knowledge on oral health among the institutionalized elderly population along with the associated factors. It demonstrates the implications for oral care by controlling or reducing influencing factors to improve oral health. Therefore, the findings of this review increase the awareness of healthcare policymakers and health promotion teams regarding oral health. This report also unveiled the current lack of standardized approaches regarding study design, data collection and analysis. More importantly, it shows the lack of longitudinal studies and RCTs. Nevertheless, this review assisted in identifying and confirming the at-risk institutionalized elderly population, providing a background for the development of strategies that will ultimately target the risk factors to potentially improve their OHRQoL, refining oral care for this specific at-risk, deprived population.

## Figures and Tables

**Table 1:** Methodological quality for cross-sectional studies.

No.	Authors	Title	1. Were the Criteria for Inclusion in the Sample Clearly Defined?	2. Were the Study Subjects and the Setting Described in Detail?	3. Was the Exposure Measured in a Valid and Reliable Way?	4. Were Objective, Standard Criteria Used for Measurement of the Condition?	5. Were Confounding Factors Identified?	6. Were Strategies to Deal with Confounding Factors Stated?	7. Were the Outcomes Measured in a Valid and Reliable Way?	8. Was Appropriate Statistical Analysis Used?	Total Score *n*/16	Level of Quality
1	Cocco, et al. (2018) [34]	The burden of tooth loss in Italian elderly population living in nursing homes.	N	N	UC	Y	N	N	UC	UC	5	Weak
2	Cornejo et al. (2013) [36]	Oral health-related quality of life in institutionalized elderly in Barcelona (Spain).	Y	Y	Y	Y	Y	Y	Y	UC	15	Strong
3	Hopcraft et al. (2012) [38]	Edentulism and dental caries in Victorian nursing homes.	N	N	Y	Y	N	N	Y	Y	8	Mod
4	Janssens et al. (2017) [32]	Medication use and its potential impact on the oral health status of nursing home residents in Flanders (Belgium).	UC	UC	Y	Y	N	N	Y	Y	8	Mod
5	Kotzer et al. (2012) [40]	Oral health-related quality of life in an aging Canadian population.	Y	Y	Y	Y	Y	Y	Y	Y	16	Strong
6	Kshetrimayum et al. (2011) [21]	Oral health-related quality of life and nutritional status of institutionalized elderly population aged 60 years and above in Mysore City, India.	Y	N	Y	Y	Y	Y	Y	Y	14	Strong
7	Mozafari, et al. (2012) [25]	Prevalence of oral mucosal lesions in institutionalized elderly people in Mashhad, Northeast Iran.	Y	Y	Y	Y	N	N	N	N	8	Mod
8	Özkan, et al. (2016) [29]	Oral health status of elderly residents in a nursing home: cross-sectional. Analytical study in a western city in Turkey.	Y	N	Y	Y	N	N	Y	UC	9	Mod
9	Philip, et al. (2012a) [39]	Oral hygiene care status of elderly with dementia and in residential aged care facilities.	Y	N	Y	Y	N	N	Y	UC	9	Mod
10	Piuvezam & de Lima (2012) [42]	Self-perceived oral health status in institutionalized elderly in Brazil.	Y	Y	Y	Y	Y	Y	Y	Y	16	Strong
11	Porter et al (2015) [37]	The impact of oral health on the quality of life of nursing home residents.	Y	Y	Y	Y	Y	Y	Y	Y	16	Strong
12	Rabiei et al. (2010) [24]	Prevalence of oral and dental disorders in institutionalized elderly people in Rasht, Iran.	Y	N	Y	Y	Y	Y	N	UC	11	Mod
13	Rekhi, et al. (2018) [22]	Periodontal status and oral health-related quality of life in elderly residents of aged care homes in Delhi.	Y	N	Y	Y	N	N	Y	Y	10	Mod
14	Saarela et al. (2014) [33]	Dentition status, malnutrition and mortality among older service housing residents.	UC	UC	Y	Y	Y	Y	Y	Y	14	Strong
15	Santucci & Attard (2015) [35]	The oral health-related quality of life in state institutionalized older adults in Malta.	Y	N	Y	Y	N	N	Y	UC	9	Mod
16	Shivakumar, et al. (2018) [23]	Oral health-related quality of life of institutionalized elderly in Satara District, India.	Y	Y	Y	Y	Y	Y	Y	UC	15	Strong
17	Takeuchi et al. (2015) [27]	Posterior teeth occlusion associated with cognition function in nursing home older residents: A cross-sectional observational study.	Y	Y	Y	Y	Y	Y	Y	Y	16	Strong
18	Tan et al. (2014) [26]	Risk indicators for root caries in institutionalized elders.	Y	Y	Y	Y	Y	Y	Y	Y	16	Strong
19	Uludamar et al. (2011) [28]	Oral health status and treatment requirements of different residential homes in Istanbul: A comparative study.	Y	Y	Y	Y	N	N	UC	UC	10	Mod
20	Zenthöfer et al (2014) [30]	Determinants of oral health-related quality of life of the institutionalized elderly.	UC	N	Y	Y	Y	Y	Y	Y	13	Strong
21	Ziebolz et al. (2017) [31]	Oral health and nutritional status in nursing home residents-results of an explorative cross-sectional pilot study.	Y	Y	Y	Y	Y	Y	Y	Y	16	Strong
22	Zimmerman et al. (2017) [41]	Readily identifiable risk factors of nursing home residents’ oral hygiene: dementia, hospice, and length of stay.	Y	N	Y	Y	Y	Y	UC	UC	12	Strong

Y: Yes = 2; UC: Unclear = 1; N: No = 0; NA: Not applicable = 0; Weak: 0–5; Moderate (Mod): 6–11; Strong: 12 or above.

**Table 2:** Methodological quality for case control studies.

No.	Authors	Title	1. Were the Groups Comparable Other Than the Presence of Disease in Cases or the Absence of Disease in Controls?	2. Were Cases and Controls Matched Appropriately?	3. Were the Same Criteria Used for Identification of Cases and Controls?	4. Was Exposure Measured in a Standard, Valid and Reliable Way?	5. Was Exposure Measured in the Same Way for Cases and Controls?	6. Were Confounding Factors Identified?	7. Were Strategies to Deal with Confounding Factors Stated?	8. Were Outcomes Assessed in a Standard, Valid and Reliable Way for Cases and Controls?	9. Was the Exposure Period of Interest Long Enough to Be Meaningful?	10. Was Appropriate Statistical Analysis Used?	Total Score (n/20)	Level of Quality
1	Brukiené et al. (2011) [44]	Salivary factors and dental plaque levels in relation to the general health of elderly residents in a long-term care facility: a pilot study	Y	N	N	Y	Y	N	N	N	N	UC	7	Mod
2	Kim, et al. (2009) [43]	Chewing function impacts oral health-related quality of life among institutionalized and community-dwelling Korean elders.	Y	Y	Y	Y	Y	N	N	Y	N	UC	13	Mod
3	Niesten et al. (2016) [45]	Oral health-related quality of life and associated factors in a care-dependent and a care-dependent older population.	Y	Y	Y	Y	Y	Y	Y	Y	N	Y	18	Strong

Y: Yes = 2; UC: Unclear = 1; N: No = 0; NA: Not applicable = 0; Weak: 0–6; Moderate (Mod): 7–13; Strong: 14 or above.

## Supplementary Materials

The following are available online at https://www.mdpi.com/1660-4601/16/21/4132/s1, Appendix 1: Comment protocols used in older population group oral health surveys, Appendix 2: Lists of included studies, Appendix 3: Additional results concerning functional ability, nutritional status, health-related quality of life and oral health, Table S1. PRISMA checklist adopted from Moher et al. 2009, Table S1: PRISMA checklist adopted from Moher et al. 2009, Table S2: EMBASE (Ovid) search strategy—30 June 2019, Table S3: CINAHL Complete (EBSCOhost) search strategy—June 30, 2019, Table S4: MEDLINE (OvidSP) search strategy—30 June 2019, Table S5: Details of excluded studies (*n* = 55), Table S6: Details of all included studies listed under continent of origin in chronological order.

## Abbreviations

ADL	Activity of Daily Living
BMI	Basic Mass Index
CASP	Critical Appraisal Skills Program
CCI	Charlson Comorbidity Index
CDR	Clinical Dementia Rating Scale
CPI	Community Periodontal Index
CPITN	Community Periodontal Index of Treatment Needs
DHI	Denture Hygiene Index
DMFT	Decayed Missing Filled Teeth
DPI	Denture Plaque Index
DT	Decayed Tooth
FT	Filled Tooth
FTU	Functional Tooth Units
GBI	Gingival Bleeding Index
GI-LTC	Gingival Index for Long-Term Care
GOHAI	General or Geriatric Oral Health Assessment Index
JBI	Joanna Briggs Institute
LOA	Loss of Attachment
MCI	Mild Cognitive Impairment
MMSE	Mini-Mental State Examination
MNA	Mini Nutritional Assessment
MT	Missing Tooth
OHRQoL	Oral Health-related Quality of Life
OHIP	Oral Health Impacts Profile
OIDP	Oral Impact on Daily Performance
PI-LTC	Plaque Index for Long-Term Care
POH	Poor Oral Health
PRISMA	Preferred Reporting Items for Systematic Reviews and Meta-analysis
PSR	Periodontal Screening and Recording
RCI	Root Caries Index
ROAG	Revised Oral Assessment Guide
SOH	Satisfactory Oral Health
TMI	Temporomandibular Joint
VPI	Visual Plaque Index
WHO	World Health Organization
