# Supplementary material for: Oral Health and Its Associated Factors Among Older Institutionalized Residents—A Systematic Review
Source: Int J Environ Res Public Health. 2019 Oct 26;16(21):4132. doi: 10.3390/ijerph16214132 (PMC6861909; doi:10.3390/ijerph16214132)
Supplement: Supplementary file 1 [file ijerph-16-04132-s001.pdf]

International Journal of
Environmental Research and Public Health

MDPI

Oral Health and Associated Factors among Older Institution Residents: A Systematic Review

	Page
**Appendix 1.** Comment protocols used in older population group oral health surveys	2
**Appendix 2.** Lists of included studies	4
**Appendix 3.** Additional results concerning functional ability, nutritional status, health-related quality of life, and oral health	6
**Table S1.** PRISMA checklist adopted from Moher et al. 2009	9
**Table S2.** EMBASE (Ovid) search strategy – June 30, 2019	12
**Table S3.** CINAHL Complete (EBSCOhost) search strategy – June 30, 2019	14
**Table S4.** MEDLINE (OvidSP) search strategy – June 30, 2019	16
**Table S5.** Details of excluded studies (n=55)	19
**Table S6.** Details of all included studies listed under continent of origin in chronological order.	24

Appendix 1. Comment protocols used in older population oral health surveys

Established epidemiological instruments are commonly used to evaluate oral conditions, in population/community studies such as the Community Periodontal Index (CPI) and Loss of Attachment (LOA) [1], the Decayed Missing Filled Teeth (DMFT) Index [1], the Denture Hygiene Index (DHI) [2], the Denture Plaque Index (DPI) [3], Gingival Bleeding Index (GBI) [4], the Gingival Index for Long-Term Care (GI-LTC) [3], Periodontal Screening and Recording (PSR) [5], the Plaque Index for Long-Term Care (PI-LTC) [3], the Revised Oral Assessment Guide (ROAG) [6], and the Visible Plaque Index (VPI) [7]. These instruments facilitate objective and comprehensive evaluation of oral health.

1.1. Oral health/hygiene examination

VPI evaluates the homecare/oral hygiene of an individual via reporting the presence and quantity of dental plaque at facial/buccal and lingual non-restored surface of all the teeth except third molars [7]. GBI is used to evaluate/categorize severity of gum inflammation/bleeding [4]. PI-LTC and GI-LTC are used to evaluate plaque control/homecare, or gingivitis (i.e. gum inflammation) in individuals under long-term care facility, respectively [3]. DPI and DHI are used to evaluate the plaque, and/or dirt/calculus accumulation on removable dentures, respectively [3].

1.2. Periodontal/gum health examination

CPI developed by the World Health Organization is used to evaluate periodontal health/disease conditions like bleeding on probing, calculus or periodontal pockets at index teeth (first and second molars and upper right/lower left central incisors) in one's mouth where dental arches are divided into sextants [1]. CPI was evolved from an older version of the same tool: The Community Periodontal Index of Treatment Needs (CPITN) [8]. Through the CPI examination, the periodontal condition is segregated into 5 categories, excluded sextant and not recorded. The highest score of 4 indicates the most severe condition [9]. Later, modification of CPI protocol was advocated to aid general dentists to efficiently and effectively triage patients with mild versus moderate to severe periodontitis [9]. A modified/refined approach, i.e. PSR was later applied to cross-sectional surveys/epidemiological studies [10]. LOA reports history of periodontal diseases via categorization of various measurements in millimeter the destruction of periodontal support i.e. loss of periodontal attachment or the distance from cemento-enamel junction to the apical extent of the gingival sulcus/periodontal pocket upon standardized probing [9].

1.3. Dental health examination

DMFT index evaluates the dentition/dental status in terms of decayed, missing and/or filled condition due to dental caries [1].

1.4. Comprehensive oral health index

ROAG assesses saliva, swallowing, speech, caries, mucosa, gums, denture retention, denture fitting and denture conditions (0 = healthy, or1 = treatment needed) [6].

1.5. Oral health related quality of life

GOAHI consists of 12 questions related to symptoms: physical functions (i. limit in kinds of food; ii. trouble biting or chewing; iii. able to swallow comfortably; iv. unable to speak clearly), pain/discomfort (v. able to eat without discomfort; viii. used medication to relieve pain; xii. sensitive to hot, cold or sweet foods), and psychosocial aspects (vi. limited contact with people; vii. pleased with look of teeth; ix. worried about teeth, gums or dentures; x. self-conscious of teeth, gums or dentures; xi. uncomfortable eating in front of others) with regard to an individual's oral health [11]. A 5-point Likert scale from 1 to 5 (never, seldom, sometimes, often, very often, or always) was adopted, with total scores ranging from 12 to 60 (reverse score for items i, ii, iv, vi, viii-xii). A higher

GOHAI indicates a better OHRQoL [11]. The summary scores or the so called ADD-GOHAI was from time to time dichotomized at ≥57 or below to categorize if someone is with high vs. low/moderate perception of oral health [12].

OHIP assesses how oral problem affects seven conceptual dimensions of OHRQoL impact: functional limitation, physical pain, psychological discomfort, physical disability, psychological disability, social disability, and handicap [13]. Severity of the impact to individual items (7 under each category in full version; 2 under each category in shortened version) was recorded using a 5-point scale (0: never/not applicable; 1: hardly ever; 2: occasionally; 3: fairly often; or 4: very often) [13]. A numeric weight can be applied on subscale score which reflect both the frequency of each impact and lay judgments about the severity of the impact [14]. Higher OHIP score indicated poorer OHRQoL.

OIDP is used to assess how oral disease/problem impact upon eight daily performances: eating and enjoying food; speaking and pronouncing words clearly; cleaning teeth; sleeping and relaxing; smiling, laughing and showing teeth without embarrassment; maintaining usual emotional state without being irritable; carrying out major work or social role; and enjoying contact with people [15]. For each daily performance, frequency and severity scores were given, ranging from 0 (lowest frequency and severity) to 5 (highest frequency and severity). For OIDP scoring, each performance score was calculated by multiplying the frequency score with the corresponding severity score. The sum of all the performance scores was divided by the maximum possible score (8 performances × 5 frequency score × 5 severity score = 200) and multiplied by 100 to give a percentage score, ranging from 0 to 100. Higher OIDP score indicated poorer OHRQoL.

References

1. World Health Organization (WHO). Oral Health Surveys: Basic Methods. Geneve: WHO **2013**. Available online: www.who.int/oral_health/publications/9789241548649/en/ (Accessed on 2 February 2019).
2. Wefers K.P. The 'Denture Hygiene Index' (DHI). *Dental Forum* **1999**, *1*, 13–15.
3. Sloane, P.D.; Zimmerman, S.; Chen, X.; Barrick, A.L.; Poole, P.; Reed, D.; Mitchell, M.; Cohen, L.W. Effect of a person-centered mouth care intervention on care processes and outcomes in three nursing homes. *J. Am. Geriatr. Soc.* **2013**, *61*, 1158-1163. doi: 10.1111/jgs.12317.
4. Ainamo, J.; Bay, I. Problems and proposals for recording gingivitis and plaque. *Int. Dent. J.* **1975**, *25*(4), 229-235. PMID: 1058834.
5. Van der Velden, U. The Dutch periodontal screening index validation and its application in The Netherlands. *J. Clin. Periodontol.* **2009**, *36*, 1018-1024. doi: 10.1111/j.1600-051X.2009.01495.x.
6. Andersson, P.; Halberg, I.R.; Renvert, S. Inter-rater reliability of an oral assessment guide for elderly patients residing in a rehabilitation ward. *Spec. Care Dentist*. **2002**, *22*, 181–186. doi: 10.1111/j.1754-4505.2002.tb00268.x.
7. Matthijs, S.; Sabzevar, M.M.; Adriaens, P.A. Intra-examiner reproducibility of 4 dental plaque indices. *J. Clin. Periodontol.* **2001**, *28*, 250-254. doi: 10.1034/j.1600-051x.2001.028003250.x.
8. World Health Organization (WHO). Oral health surveys: basic methods. 3rd edition. Geneva, Switzerland. **1987**, ISBN: 9241542160.
9. Jeffcoat, M.K.; McGuire, M.; Newman, M.G. Evidence-based periodontal treatment. Highlights from the 1996 World Workshop in Periodontics. *J. Am. Dent. Assoc.* **1997**, *128*, 713-724. doi: 10.14219/jada.archive.1997.0294.
10. Covington, L.L.; Breault, L.G.; Hokett, S.D. The application of periodontal screening and recording™ (PSR) in a military population. *J. Contemp. Dent. Pract*. **2003**, *4*, 36-51. doi: 10.5005/jcdp-4-3-24.
11. Atchison, K.A.; Dolan, T.A. Development of the Geriatric Oral Health Assessment Index. *J. Dent. Educ.* **1990**, *54*, 680–687. PMID: 2229624.
12. El Osta, N.; Tubert-Jeannin, S.; Hennequin, M.; Bou Abboud Naaman, N.; El Osta, L.; Geahchan, N. Comparison of the OHIP-14 and GOHAI as measures of oral health among elderly in Lebanon. *Health Qual. Life Outcomes* **2012**, *10*, 131. doi: 10.1186/1477-7525-10-131.

13. Slade, G.D.; Spencer, A.J. Development and evaluation of the Oral Health Impact Profile. *Community Dental Health* **1994**, *11*, 3-11. PMID: 8193981.
14. Locker D. Concepts of oral health, disease and the quality of life. In: Slade GD, editor. Measuring oral health and quality of life. Chapel Hill: University of North Carolina: Dental Ecolog; 1997, pp. 11–24.
15. Adulyanon, S.; Vourapukjaru, J.; Sheiham, A. Oral impacts affecting daily performance in a low dental disease Thai population. *Community Dent. Oral Epidemiol.* **1996**, *24*, 385-389. doi: 10.1111/j.1600-0528.1996.tb00884.x

Appendix 2. Lists of included studies in alphabetical order of authors

1. Brukienė, V.; Aleksejūnienė, J.; Gairionyte, A.; Salivary factors and dental plaque levels in relation to the general health of elderly residents in a long-term care facility: a pilot study. *Spec. Care Dentist.* **2011**, *31*, 27-32. [44]
2. Cocco, F.; Campus, G.; Strohmenger, L.; Ardizzone, V.C.; Cagetti, M.G. The burden of tooth loss in Italian elderly population living in nursing homes. *BMC Geriatrics* **2018**, *18*, 76. [34]
3. Cornejo, M.; Pérez, G.; de Lima, K.C.; Casals-Peido, E.; Borrell, C. Oral health-related quality of life in institutionalized elderly in Barcelona (Spain). *Med. Oral Patol. Oral Cir. Bucal.* **2013**, *18*, e285-e292. [36]
4. Hopcraft, M.S.; Morgan, M.V.; Satur, J.G.; Wright, F.A.C. Edentulism and dental caries in Victorian nursing homes. *Gerodontology* **2012**, *29*, e512-e519. [38]
   The report below was referred to because it shared the same study sample:
   - Hopcraft, M.S.; Morgan, M.V.; Satur, J.G.; Wright, F.A.C.; Darby, I.B. Oral hygiene and periodontal disease in Victorian nursing homes. *Gerodontology* **2012**, *29*, e220-228. doi: 10.1111/j.1741-2358.2010.00448.x. [58]
5. Janssens, B.; Petrovic, M.; Jacquet, W.; Schols, J. M.; Vanobbergen, J.; De Visschere, L. Medication use and its potential impact on the oral health status of nursing home residents in Flanders (Belgium). *J. Am. Med. Dir. Assoc*. **2017**, *18*(9), 1.e1-8. [32]
   The report below was referred to because it shared the same study sample:
   - Janssens, B.; Vanobbergen, J.; Petrovic, M.; Jacquet, W.; Schols, J.M.G.A.; De Visschere, L. The oral health condition and treatment needs of nursing home residents in Flanders (Belgium). *Community Dent. Health* **2017**, *34*, 143-151. [57]
6. Kim, H.Y.; Jang, M.S.; Chung, C.P.; Paik, D.I.; Park, Y.D.; Patton, L.L.; Ku, Y. Chewing function impacts oral health-related quality of life among institutionalized and community-dwelling Korean elders. *Community Dent. Oral Epidemiol*. **2009**, *37*, 468-476. [43]
7. Kotzer, R. D.; Lawrence, H. P.; Clovis, J. B.; Matthews, D. C. Oral health-related quality of life in an aging Canadian population. *Health Qual. Life Outcomes* **2012**, *10*, 50. [40]
   The report below was referred to because it shared the same study sample:
   - Matthews, D. C.; Clovis, J. B.; Brillant, M. G.; Filiaggi, M. J.; McNally, M. E.; Kotzer, R. D.; Lawrence, H. P. Oral health status of long-term care residents-a vulnerable population. *J. Can. Dent. Assoc*. **2012**, *78*(3), c3. [60]
8. Kshetrimayum, N.; Reddy, C.V.K.; Siddhana, S.; Manjunath, M.; Rudraswamy, S.; Sulavai, S. Oral health-related quality of life and nutritional status of institutionalized elderly population aged 60 years and above in Mysore City, India. *Gerodontology* **2013**, *30*, 119-125. doi: 10.1111/j.1741-2358.2012.00651.x [21]
9. Mozafari, P.M.; Dalirsani, Z.; Delavarian, Z.; Amirchaghmaghi, M.; Shakeri, M.T.; Esfandyari, A.; Falaki, F. Prevalence of oral mucosal lesions in institutionalized elderly people in Mashhad, Northeast Iran. *Gerodontology* **2012**, *29*, e930-e934. [25]
10. Niesten, D.; Witter, D.; Bronkhorst, E.; Creugers, N. Oral health-related quality of life and associated factors in a care-dependent and a care-dependent older population. *J. Dent.* **2016**, *55*, 33-39. [45]
    The report below was referred to because it shared the same study sample:

- Niesten, D.; Witter, D.; Bronkhorst, E.; Creugers, N. Oral health care behavior and frailty-related factors in a care-dependent older population. *J. Dent*. **2017**, *61*, 39-47. [56]

11. Özkan, G.; Arikan, A., Abacigil, F.; Pirinççi, S. Oral health status of elderly residents in a nursing home: cross-sectional. Analytical study in a western city in Turkey. *Turkish J. Geriatrics* **2016**, *19*, 87-94. [29]
12. Philip, P.; Rogers, C.; Kruger, E.; Tennant, M. Oral hygiene care status of elderly with dementia and in residential aged care facilities. *Gerodontology* **2012**, *29*, e306-e311. [39]
The report below was referred to because it shared the same study sample:
    - Philip, P.; Rogers, C.; Kruger, E.; Tennant, M. Caries experience of institutionalized elderly and its association with dementia and functional status. *Int. J. Dent. Hyg.* ***2012***, *10*, 122-127. [59]
13. Piuvezam, G.; de Lima, K.C. Self-perceived oral health status in institutionalized elderly in Brazil. *Arch. Gerontol. Geriatr*. **2012**, *55*, 5-11. [42]
The reports below were referred to because they shared the same study sample:
    - de Mederiros, A.K.B.; Barbosa, F.P.; Pivuvezam, G.; Carreiro, A.D.F.P.; Lime, K.C. Prevalence and factors associated with alterations of the temporomandibular joint in institutionalized elderly. *Cien. Saude Colet*. **2019**, *24*,159-168. [62]
    - Piuvezam, G.; de Lima, K.C. Factors associated with missing teeth in the Brazilian elderly institutionalized population. *Gerodontology* **2013**, *30*, 141-149. doi: 10.1111/j.1741-2358.2012.00655x. [61]
14. Porter, J.; Ntouva, A.; Read, A.; Murdoch, M.; Ola, D.; Tsakos, G. The impact of oral health on the quality of life of nursing home residents. *Health and Quality of Life Outcomes* **2015**, *13*, 102. doi:10.1186/s12955-015-0300-y. [37]
15. Rabiei, M.; Kasemnezhad, E.; Masoudi rad, H.; Shakiba, M.; Pourkay, H. Prevalence of oral and dental disorders in institutionalized elderly people in Rasht, Iran. *Gerodontology* **2010**, *27*, 174-177. [24]
16. Rekhi, A.; Marya, C.M.; Oberoi, S.S.; Nagpal, R.; Dhingra, C.; Kataria, S. Periodontal status and oral health-related quality of life in elderly residents of aged care homes in Delhi. *Geriatr. Gerontol. Int.* **2016**, *16*, 474-480. [22]
The report below was referred to because it shared the same study sample:
    - Rekhi, A.; Marya, C.M.; Nagpal, R.; Oberoi, S.S. Assessment of oral health related quality of life among the institutionalized elderly in Delhi, India. *Oral Health Prev. Dent.* **2018**, *16*, 59-66. [46]
17. Saarela, R. K.; Soini, H.; Hiltunen, K.; Muurinen, S.; Suominen, M.; Pitkala, K. Dentition status, malnutrition and mortality among older service housing residents. *J. Nutr. Health Aging* **2014**, *18*, 34-38. [33]
The report below was referred to because it shared the same study sample:
    - Saarela, R.K.T.; Lindroos, E.; Soini, H.; Hiltunen, K.; Muurinen, S.; Suominen, M.; Pitkala, K. Dentition, nutritional status and adequacy of dietary intake among older residents in assisted living facilities. *Gerodontology* **2016**, *33*, 225-232. [49]
18. Santucci, D.; Attard, N. The oral health-related quality of life in state institutionalized older adults in Malta. *Int. J. Prosthodont*. **2015**, *28*, 402-411. [35]
The report below was referred to because it shared the same study sample:
    - Santucci, D.; Attard, N. The oral health of institutionalized older adults in Malta. *Int.J. Prosthodont*. **2015,** *28*, 146-148. [55]
19. Shivakumar, K.; Patil, S.; Kadashetti, V.; Raje, V. Oral health-related quality of life of institutionalized elderly in Satara District, India. *J. Datta Meghe. Inst. Med. Sci. Univ.* **2018**, *13*, 183-189. [23]
20. Takeuchi, K.; Izumi, M.; Furuta, M.; Takeshita, T.; Shibata, Y.; Kageyama, S.; Ganaha, S.; Yamashita, Y. Posterior teeth occlusion associated with cognition function in nursing home older residents: A cross-sectional observational study. *PLoS ONE* **2015**, *10*, e0141737. [27]
The report below was referred to because it shared the same study sample:

- Takeuchi, K.; Izumi, M.; Furuta, M.; Takeshita, T.; Shibata, Y.; Kageyama, S.; Ganaha, S.; Yamashita, Y. Association between posterior teeth occlusion and functional dependence among older adults in nursing homes in Japan. *Geriatr. Gerontol. Int.* **2017**, *17*, 622-627. [47]

21. Tan, H. P.; Lo, E. C. Risk indicators for root caries in institutionalized elders. *Community Dent. Oral Epidemiol.* **2014**, *42*, 435-440. [26]
22. Uludamar, A.; Evren, B.A.; Işeri, U.; Özkan, Y.K. Oral health status and treatment requirements of different residential homes in Istanbul: A comparative study. *Arch. Gerontol. Geriat.* **2011**, *53*, e67-e74. [28]
    The report below was referred to because it shared the same study sample:
    - Evren, B.A.; Uludamar, A.; Işeri, U.; Özkan, Y.K. The association between socioeconomic status, oral hygiene practice, denture stomatitis and oral status in elderly people living different residential homes. *Arch. Gerontol. Geriat.* **2011**, *53*, 252-257. [48]
23. Zenthöfer, A.; Rammelsberg, P.; Cabrera, T.; Schroder, J.; Hassel, A.J. Determinants of oral health-related quality of life of the institutionalized elderly. *Psychogeriatrics* **2014**, *14*, 247–254. [30]
    The reports below were referred to because they shared the same study sample:
    - Zenthöfer, A.; Rammelsberg, P.; Cabrera, T.; Hassel, A.J. Increasing dependency of older people in nursing homes is associated with need for dental treatments. *Neuropsychiatr. Dis. Treat.* **2014**, *10*, 2285-2290. [50]
    - Zenthöfer, A.; Schröder, R.P.; Cabrera, T.; Rammelsberg, P.; Hassel, A.J. Comparison of oral health among older people with and without dementia. *Community Dent. Health* **2014**, *31*, 27-31. [51]
    - Zenthöfer, A.; Navratil, S.D.; Rammelsberg, P.; Cabrera, T.; Gorenc, L.; Urbanowitsch, N.; Wetzel, P.; Schröder, J.; Hassel, A.J. Oral health and apraxia among institutionalized elderly people – A pilot study. *Acta Odontol. Scand.* **2015**, *73*, 150-155. [52]
    - Zenthöfer, A.; Baumgart, D.; Cabrera, T.; Rammelsberg, P.; Schröder, J.; Corcodel, N.; Hassel, A.J. Poor dental hygiene and periodontal health in nursing home residents with dementia: an observational study. *Odontology* **2017**, *105*, 208-213. [53]
    - Klotz, A.L.; Hassel, A.J.; Schröder, R.P.; Zenthöfer, A. Oral health-related quality of life and prosthetic status of nursing home residents with or without dementia. Clin. Interv. *Aging* **2017**, *12*, 659-665. [54]
24. Ziebolz, D.; Werner, C.; Schmalz, G.; Nitschke, L.; Haak, R.; Mausberg, R.F.; Chenot, J.F. Oral health and nutritional status in nursing home residents-results of an explorative cross-sectional pilot study. *BMC Geriatrics* **2017**, *17*, 39. doi: 10.1186/s12877-017-0429-0. [31]
25. Zimmerman, S.; Austin, S.; Cohen, L.; Reed, D.; Poole, P.; Ward, K.; Sloane, P.D. Readily identifiable risk factors of nursing home residents' oral hygiene: dementia, hospice, and length of stay. *J. Am. Geriatr. Soc.* **2017**, *65*, 2516-2521. [41]

Appendix 3. Additional results concerning functional ability, nutritional status, health-related quality of life, and oral health

3.1. Functional ability

Functional ability of the institutionalized elderly surveyed was evaluated using various methods in five included studies [27, 30, 32, 33, 45] in this review. In brief, Barthel Index (BI) was used to assess the functional capacity or dependency of the residents surveyed in two studies [27, 30]. Only the Japanese study [27] reported 14.1% of their residents surveyed were considered functionally independent. The German study reported the mean BI score only. Saarela et al. [33] used Clinical Dementia Rating scale (CDR) personal care subscale (>2) to gauge physical functioning dependency and reported that 68.4% of the 1,369 Finish residents surveyed were functionally dependent. Niesten

et al. [45] used Gobbens' definition of frailty scale to characterize the 126 residents in their report and identified approximate 1/3 each of their residents were medium or high care-dependent while 1/8 were of low care-dependency. Janssens et al. [32] used Activity of Daily Living (ADL) scale to gauge the functional ability of their 1,226 residents surveyed, and reported >50%, >25% and approximate 20% of the residents surveyed were of high, medium or low dependency, respectively.

One study [39] investigated the Activities of Daily Oral Hygiene (Bauer 2001) in a modified approach (mADOH) and reported slightly more than half of the 193 surveyed residents were self-oral hygiene/care disable, while around 1/5 each were partially able or able.

3.2. Nutritional status

Four included reports [21, 31, 33, 34] characterized the nutritional status of their surveyed residents using the Mini Nutritional Assessment (MNA) tool or the short form version (MNA-SF). An Indian survey [21] reported that only 1/3 of the 141 residents followed were adequately nourish while >50%, or one out of six residents were at risk of, or actually malnourished, respectively. A Finish study [33] indicated approximately 1/8, 2/3 and 1/5 of the 1,369 residents surveyed were malnourished, at risk of malnourish, or well nourished, respectively. A German study [31] reported that 51.7% of their 87 surveyed residents were 'at risk of malnutrition' (MNA 8-11) while 48.3% were of 'normal' (MNA 12-14) nutritional status and none malnourished. The data presented, however, was categorized in a way inconsistent with the standard protocol mentioned in the methods section of the same paper. An Italian national study [34] indicated 5.7%, 40.3%, 54.0% of 741 residents surveyed were malnourished, at risk of malnourish, or well nourished, respectively.

3.3. Health-related quality of life (HRQoL) and perceived social support

Only one included study from the Netherlands [45] reported the HRQoL, social support of the 126 institutionalized elderly followed using 12-Item Short Form Survey (SF-12) questionnaire, or ENRICHED Social Support Index (ESSI), respectively. Dutch age- and sex-standardized norm scores for 70-79 years (SF-12 physical component summary: 44.1; SF-12 mental component summary: 49.5) were used as reference that any score higher than the norm indicate better HRQoL. Niesten et al. [45] reported 17.5%, 50.8% of the institutionalized elderly surveyed had physical or mental HRQoL component ≥the population norm, respectively while 26.2%, 38.9% 34.1% experienced low, medium, or high social support.

3.4. Addition oral health results

3.4.1. Dental attendance

Seven studies [23, 28, 38-42] reported the frequency of dental visits of the institutionalised elderly surveyed. In six of fourteen (42.9%) nursing homes from a United States study [41], dental services were available. Similarly, high percentage of long-term care residents (75.3%) reporting <1 dental visit per year was stated in a Canadian study [40]. The other reports, however, indicated a different scenario. A Turkish study [28] reported 38% of surveyed residents had had dental visits ≤1 year driven by oral symptoms. A later report from the same country [39] indicated that only 4.4% of residents studied had visited dentist regularly and about 70% were irregular attender. An Indian study reported also <9% of their older institution residents surveyed visited a dentist in past one year [23]. An Australian study [38] reported only 25% residents had dental visit in past two years which was similar to a Brazilian report [42] indicating 21.8% residents had dental visit within past 12 months.

3.4.2. Oral mucosal conditions

Six studies [22, 25, 27, 33, 43, 44] comprehensively evaluated oral mucosal conditions of institutionalised elderly without explicitly indicate if standardized assessment tool/protocol was used.

3.4.3. Temporomandibular joint status

de Medeiro and co-workers [62] reported using the same resident sample in Piuvezam and de Lima [42], the temporomandibular joint (TMJ) status of the Brazilian surveyed. No detail was given regarding how the TMJs were assessed.

3.4.4. Salivary flow, pH and buffer capacity

Salivary flow rate, pH and buffer capacity were assessed by Brukiené et al. [44] on a cohort of Lithuanian institutionalized elderly while prevalence of dry mouth was reported in four papers [22, 25, 33, 37]. Janssens et al. [32] profiled the medications potentially causing dry mouth among the Belgian residents surveyed.

Reference

Please refer to reference list of main text.

Bauer, J.G. The index of ADOH: Concept of measuring oral self-care functioning in the elderly. *Spec. Care. Dentist.* **2001**, *21*, 63–67. doi: 10.1111/j.1754-4505.2001.tb00227.x

Table S1. PRISMA checklist adopted from Moher et al. 2009.

Section/Topic	Item no.	Checklist items	Section	Page
**TITLE**				
Title	1	Identify the report as a systematic review, meta-analysis, or both.	Title	1
**ABSTRACT**				
Structured summary	2	Provide a structured summary including, as applicable: background; objectives; data sources; study eligibility criteria, participants, and interventions; study appraisal and synthesis methods; results; limitations; conclusions and implications of key findings; systematic review registration number.	Abstract	1
**INTRODUCTION**				
Rationale	3	Describe the rationale for the review in the context of what is already known.	Introduction	1-2
Objectives	4	Provide an explicit statement of questions being addressed with reference to participants, interventions, comparisons, outcomes, and study design (PICOS).	Methods	2
**METHODS**				
Protocol and registration	5	Indicate whether a review protocol exists, if and where it can be accessed (eg., Web address), and, if available, provide registration information including registration number.	Not exist	-
Eligibility criteria	6	Specify study characteristics (e.g., PICOS, length of follow-up) and report characteristics (e.g., years considered, language, publication status) used as criteria for eligibility, giving rationale.	Methods	2-3
Information sources	7	Describe all information sources (e.g., databases with dates of coverage, contact with study authors to identify additional studies) in the search and date last searched.	Methods & Supplementary files	2-4, Appendix 1-3, Table S1-4, Table 3
Search	8	Present full electronic search strategy for at least one database, including any limits used, such that it could be repeated.	Supplementary files	3-4; Appendix 2 & 3, Table S1-4
Study selection	9	State the process for selecting studies (i.e., screening, eligibility, included in systematic review, and, if applicable, included in the meta-analysis).	Methods & Supplementary files	2-4; Figure 1
Data collection process	10	Describe method of data extraction from reports (e.g., piloted forms, independently, in duplicate) and any processes for obtaining and confirming data from investigators.	Methods	2-4; Table 3
Data items	11	List and define all variables for which data were sought (e.g., PICOS, funding sources) and any assumptions and simplifications made.	Methods & Supplementary files	3-4, Table 3
Risk of bias in individual studies	12	Describe methods used for assessing risk of bias of individual studies (including	Methods	4; Table 1 & 2

		specification of whether this was done at the study or outcome level), and how this information is to be used in any data synthesis.		
Summary measures	13	State the principal summary measures (e.g., risk ratio, difference in means).	Methods	4, Table 1&2
Synthesis of results	14	Describe the methods of handling data and combining results of studies, if done, including measures of consistency (e.g., I^2) for each meta-analysis.	Methods	3-4, Table 3
Risk of bias across studies	15	Specify any assessment of risk of bias that may affect the cumulative evidence (e.g., publication bias, selective reporting within studies).	Methods, Results	3-4; Table 1-3
Additional analyses	16	Describe methods of additional analyses (e.g., sensitivity or subgroup analyses, meta-regression), if done, indicating which were pre-specified.	Not exist	-
**RESULTS**				
Study selection	17	Give numbers of studies screened, assessed for eligibility, and included in the review, with reasons for exclusions at each stage, ideally with a flow diagram.	Methods	5, Figure 1
Study characteristics	18	For each study, present characteristics for which data were extracted (e.g., study size, PICOS, follow-up period) and provide the citations.	Results Tables	8-10; Table 3
Risk of bias within studies	19	Present data on risk of bias of each study and, if available, any outcome level assessment (see item 12).	Results	6; Table 1 & 2
Results of individual studies	20	For all outcomes considered (benefits or harms), present, for each study: a) simple summary data for each intervention group b) effect estimates and confidence intervals, ideally with a forest plot.	Results, Supplementary files	8-19; Table 3
Synthesis of results	21	Present the main results of the review. If meta-analyses are done, include for each, confidence intervals and measures of consistency.	Results, Supplementary files	8-19, Table 3
Risk of bias across studies	22	Present results of any assessment of risk of bias across studies (see Item 15).	Results, Supplementary files	6, Table 1 & 2
Additional analysis	23	Give results of additional analyses, if done (e.g., sensitivity or subgroup analysis, meta-regression [see Item 16]).	Not exit	_
**DISCUSSION**				
Summary of evidence	24	Summarize the main findings including the strength of evidence for each main outcome; consider their relevance to key groups (e.g., healthcare providers, users, and policy makers).	Discussion	19-23, Table 3
Limitations	25	Discuss limitations at study and outcome level (e.g., risk of bias), and at review-level (e.g., incomplete retrieval of identified research, reporting bias).	Discussion	23
Conclusions	26	Provide a general interpretation of the results in the context of other evidence, and implications for future research.	Discussion	23

**FUNDING**				
Funding	27	Describe sources of funding for the systematic review and other support (e.g., supply of data); role of funders for the systematic review.	Funding	24

Table S2. EMBASE (Ovid) search strategy – June 30, 2019.

EMBASE (Ovid)		
No.	Searches	Results
1	Oral.mp. [mp=title, abstract, heading word, drug trade name, original title, device manufacturer, drug manufacturer, device trade name, keyword, floating subheading word, candidate term word]	1508878
2	dental.mp. [mp=title, abstract, heading word, drug trade name, original title, device manufacturer, drug manufacturer, device trade name, keyword, floating subheading word, candidate term word]	337518
3	1 or 2	1782671
4	health.mp. [mp=title, abstract, heading word, drug trade name, original title, device manufacturer, drug manufacturer, device trade name, keyword, floating subheading word, candidate term word]	3750426
5	problem.mp. [mp=title, abstract, heading word, drug trade name, original title, device manufacturer, drug manufacturer, device trade name, keyword, floating subheading word, candidate term word]	616856
6	4 or 5	4178514
7	3 and 6	223840
8	health-related quality of life.mp. [mp=title, abstract, heading word, drug trade name, original title, device manufacturer, drug manufacturer, device trade name, keyword, floating subheading word, candidate term word]	58902
9	1 and 8	4952
10	old.mp. [mp=title, abstract, heading word, drug trade name, original title, device manufacturer, drug manufacturer, device trade name, keyword, floating subheading word, candidate term word]	1396213
11	aged.mp. [mp=title, abstract, heading word, drug trade name, original title, device manufacturer, drug manufacturer, device trade name, keyword, floating subheading word, candidate term word]	4041494
12	10 and 11	385455
13	residential.mp. [mp=title, abstract, heading word, drug trade name, original title, device manufacturer, drug manufacturer, device trade name, keyword, floating subheading word, candidate term word]	50227
14	nursing.mp. [mp=title, abstract, heading word, drug trade name, original title, device manufacturer, drug manufacturer, device trade name, keyword, floating subheading word, candidate term word]	556803
15	long-term.mp. [mp=title, abstract, heading word, drug trade name, original title, device manufacturer, drug manufacturer, device trade name, keyword, floating subheading word, candidate term word]	1104007
16	12 or 13 or 14 or 15	2038365
17	home*.mp. [mp=title, abstract, heading word, drug trade name, original title, device manufacturer, drug manufacturer, device trade name, keyword, floating subheading word, candidate term word]	726819
18	facilit*.mp. [mp=title, abstract, heading word, drug trade name, original title, device manufacturer, drug manufacturer, device trade name, keyword, floating subheading word, candidate term word]	835279
19	institution*.mp. [mp=title, abstract, heading word, drug trade name, original title, device manufacturer, drug manufacturer, device trade name, keyword, floating subheading word, candidate term word]	430555
20	17 or 18 or 19	1891698
21	16 and 20	265294
22	factor*.mp. [mp=title, abstract, heading word, drug trade name, original title, device manufacturer, drug manufacturer, device trade name, keyword, floating subheading word, candidate term word]	5268371
23	precipitat*.mp. [mp=title, abstract, heading word, drug trade name, original title, device manufacturer, drug manufacturer, device trade name, keyword, floating subheading word, candidate term word]	148359
24	22 and 23	29930

25	predict*.mp. [mp=title, abstract, heading word, drug trade name, original title, device manufacturer, drug manufacturer, device trade name, keyword, floating subheading word, candidate term word]	2038834
26	determinant*.mp. [mp=title, abstract, heading word, drug trade name, original title, device manufacturer, drug manufacturer, device trade name, keyword, floating subheading word, candidate term word]	273566
27	22 or 24 or 25 or 26	6818090
28	decayed missing filled teeth.mp. [mp=title, abstract, heading word, drug trade name, original title, device manufacturer, drug manufacturer, device trade name, keyword, floating subheading word, candidate term word]	331
29	community periodontal index.mp. [mp=title, abstract, heading word, drug trade name, original title, device manufacturer, drug manufacturer, device trade name, keyword, floating subheading word, candidate term word]	818
30	community periodontal index of treatment needs.mp. [mp=title, abstract, heading word, drug trade name, original title, device manufacturer, drug manufacturer, device trade name, keyword, floating subheading word, candidate term word]	345
31	visual plaque index.mp. [mp=title, abstract, heading word, drug trade name, original title, device manufacturer, drug manufacturer, device trade name, keyword, floating subheading word, candidate term word]	9
32	periodontal loss of attachment.mp. [mp=title, abstract, heading word, drug trade name, original title, device manufacturer, drug manufacturer, device trade name, keyword, floating subheading word, candidate term word]	17
33	revised oral assessment guide.mp. [mp=title, abstract, heading word, drug trade name, original title, device manufacturer, drug manufacturer, device trade name, keyword, floating subheading word, candidate term word]	27
34	plaque index for long term care.mp. [mp=title, abstract, heading word, drug trade name, original title, device manufacturer, drug manufacturer, device trade name, keyword, floating subheading word, candidate term word]	4
35	gingival index for long term care.mp. [mp=title, abstract, heading word, drug trade name, original title, device manufacturer, drug manufacturer, device trade name, keyword, floating subheading word, candidate term word]	4
36	denture plaque index.mp. [mp=title, abstract, heading word, drug trade name, original title, device manufacturer, drug manufacturer, device trade name, keyword, floating subheading word, candidate term word]	11
37	periodontal screening and recording.mp. [mp=title, abstract, heading word, drug trade name, original title, device manufacturer, drug manufacturer, device trade name, keyword, floating subheading word, candidate term word]	57
38	denture hygiene index.mp. [mp=title, abstract, heading word, drug trade name, original title, device manufacturer, drug manufacturer, device trade name, keyword, floating subheading word, candidate term word]	14
39	7 or 28 or 29 or 30 or 31 or 32 or 33 or 34 or 35 or 36 or 37 or 38	224227
40	oral health impact profile.mp. [mp=title, abstract, heading word, drug trade name, original title, device manufacturer, drug manufacturer, device trade name, keyword, floating subheading word, candidate term word]	1270
41	general oral health assessment index.mp. [mp=title, abstract, heading word, drug trade name, original title, device manufacturer, drug manufacturer, device trade name, keyword, floating subheading word, candidate term word]	82
42	geriatric oral health assessment index.mp. [mp=title, abstract, heading word, drug trade name, original title, device manufacturer, drug manufacturer, device trade name, keyword, floating subheading word, candidate term word]	133
43	oral impact on daily performance.mp. [mp=title, abstract, heading word, drug trade name, original title, device manufacturer, drug manufacturer, device trade name, keyword, floating subheading word, candidate term word]	56
44	9 or 40 or 41 or 42 or 43	5531
45	39 or 44	224231

46	people.mp. [mp=title, abstract, heading word, drug trade name, original title, device manufacturer, drug manufacturer, device trade name, keyword, floating subheading word, candidate term word]	586305
47	10 and 46	42383
48	elderly.mp. [mp=title, abstract, heading word, drug trade name, original title, device manufacturer, drug manufacturer, device trade name, keyword, floating subheading word, candidate term word]	503526
49	resident*.mp. [mp=title, abstract, heading word, drug trade name, original title, device manufacturer, drug manufacturer, device trade name, keyword, floating subheading word, candidate term word]	236844
50	47 or 48 or 49	752480
51	21 and 27 and 45 and 50	564
52	limit 51 to year = '2008-current'	372
53	limit 52 to English language	360
54	limit 53 to abstracts	356
55	limit 54 to aged 65+ years	228
56	limit 55 to article	164

Table S3. CINAHL Complete (EBSCOhost) search strategy – June 30, 2019.

CINAHL Complete (EBSCOhost): Search modes – Boolean/Phrase			
Search	Search Terms	Search Options	Results
S1	oral		128975
S2	dental		104183
S3	S1 or S2		210964
S4	health		1534540
S5	problem		225769
S6	S4 or S5		1657983
S7	S3 and S6		53010
S8	health-related quality of life		18876
S9	S1 and S8		1567
S10	old		166063
S11	aged		824542
S12	S10 and S11		114900
S13	residential		19346
S14	nursing		654803
S15	long-term		163851
S16	S12 or S13 or S14 or S15		899452
S17	home*		212368
S18	facilit*		210523
S19	institution*		88330
S20	S17 or S18 or S19		474750
S21	S16 and S20		135842
S22	factor*		1326188
S23	precipitat*		5766
S24	S22 and S23		2581
S25	predict*		323044
S26	determinant*		41149
S27	S22 or S24 or S25 or S26		1504208
S28	decayed missing filled teeth		92
S29	community periodontal index		215
S30	community periodontal index of treatment needs		55
S31	visual plaque index		2
S32	periodontal loss of attachment		4
S33	revised oral assessment guide		14
S34	plaque index for long term care		3
S35	gingival index for long term care		3

S36	denture plaque index		5
S37	periodontal screening and recording		9
S38	denture hygiene index		6
S39	S7 or S28 or S29 or S30 or S31 or S32 or S33 or S34 or S35 or S36 or S37 or S38		53113
S40	oral health impact profile		454
S41	general oral health assessment index		39
S42	geriatric oral health assessment index		47
S43	oral impact on daily performance		21
S44	S9 or S40 or S41 or S42 or S43		1783
S45	S39 or S44		53114
S46	people		209937
S47	S10 and S46		21379
S48	elderly		85968
S49	resident*		67931
S50	S47 or S48 or S49		161710
S51	S21 and S27 and S45 and S50		281
S52	S21 and S27 and S45 and S50	**Limiters**<br>**Published Date:** 20080701-20190630	201
S53	S21 and S27 and S45 and S50	**Limiters**<br>**Published Date:** 20080701-20190630;<br>**Narrow by Language:** English	199
S54	S21 and S27 and S45 and S50	**Limiters**<br>**Published Date:** 20080701-20190630;<br>**Narrow by Language:** English;<br>**Abstract Available**	171
S55	S21 and S27 and S45 and S50	**Limiters**<br>**Published Date:** 20080701-20190630;<br>**Narrow by Language:** English;<br>**Abstract Available;**<br>**Narrow by Subject Age:** aged: 65+ years	122
S56	S21 and S27 and S45 and S50	**Limiters**<br>**Published Date:** 20080701-20190630;<br>**Narrow by Language:** English;<br>**Abstract Available;**<br>**Narrow by Subject Age:** aged: 65+ years;<br>**Academic Journals**	72

Table S4. MEDLINE (OvidSP) search strategy – June 30, 2019

MEDLINE (OvidSP)		
No.	Searches	Results
1	oral.mp. [mp=title, abstract, original title, name of substance word, subject heading word, floating sub- heading word, keyword heading word, organism supplementary concept word, protocol supplementary concept word, rare disease supplementary concept word, unique identifier, synonyms]	591051
2	dental.mp. [mp=title, abstract, original title, name of substance word, subject heading word, floating sub- heading word, keyword heading word, organism supplementary concept word, protocol supplementary concept word, rare disease supplementary concept word, unique identifier, synonyms]	423275
3	1 or 2	949854
4	health.mp. [mp=title, abstract, original title, name of substance word, subject heading word, floating sub- heading word, keyword heading word, organism supplementary concept word, protocol supplementary concept word, rare disease supplementary concept word, unique identifier, synonyms]	2433290
5	problem.mp. [mp=title, abstract, original title, name of substance word, subject heading word, floating sub- heading word, keyword heading word, organism supplementary concept word, protocol supplementary concept word, rare disease supplementary concept word, unique identifier, synonyms]	418901
6	4 or 5	2741528
7	3 and 6	125156
8	health-related quality of life.mp. [mp=title, abstract, original title, name of substance word, subject heading word, floating sub- heading word, keyword heading word, organism supplementary concept word, protocol supplementary concept word, rare disease supplementary concept word, unique identifier, synonyms]	33535
9	1 and 8	2605
10	old.mp. [mp=title, abstract, original title, name of substance word, subject heading word, floating sub- heading word, keyword heading word, organism supplementary concept word, protocol supplementary concept word, rare disease supplementary concept word, unique identifier, synonyms]	842015
11	aged.mp. [mp=title, abstract, original title, name of substance word, subject heading word, floating sub- heading word, keyword heading word, organism supplementary concept word, protocol supplementary concept word, rare disease supplementary concept word, unique identifier, synonyms]	5013148
12	10 and 11	336654
13	residential.mp. [mp=title, abstract, original title, name of substance word, subject heading word, floating sub- heading word, keyword heading word, organism supplementary concept word, protocol supplementary concept word, rare disease supplementary concept word, unique identifier, synonyms]	30323
14	nursing.mp. [mp=title, abstract, original title, name of substance word, subject heading word, floating sub- heading word, keyword heading word, organism supplementary concept word, protocol supplementary concept word, rare disease supplementary concept word, unique identifier, synonyms]	556919
15	long-term.mp. [mp=title, abstract, original title, name of substance word, subject heading word, floating sub- heading word, keyword heading word, organism supplementary concept word, protocol supplementary concept word, rare disease supplementary concept word, unique identifier, synonyms]	662035
16	12 or 13 or 14 or 15	1543757
17	home*.mp. [mp=title, abstract, original title, name of substance word, subject heading word, floating sub- heading word, keyword heading word, organism supplementary concept word, protocol supplementary concept word, rare disease supplementary concept word, unique identifier, synonyms]	491197
18	facilit*.mp. [mp=title, abstract, original title, name of substance word, subject heading word, floating sub- heading word, keyword heading word, organism supplementary concept word, protocol supplementary concept word, rare disease supplementary concept word, unique identifier, synonyms]	594672

19	institution*.mp. [mp=title, abstract, original title, name of substance word, subject heading word, floating sub- heading word, keyword heading word, organism supplementary concept word, protocol supplementary concept word, rare disease supplementary concept word, unique identifier, synonyms]	245016
20	17 or 18 or 19	1262393
21	16 and 20	1185149
22	factor*.mp. [mp=title, abstract, original title, name of substance word, subject heading word, floating sub- heading word, keyword heading word, organism supplementary concept word, protocol supplementary concept word, rare disease supplementary concept word, unique identifier, synonyms]	4902961
23	precipitat*.mp. [mp=title, abstract, original title, name of substance word, subject heading word, floating sub- heading word, keyword heading word, organism supplementary concept word, protocol supplementary concept word, rare disease supplementary concept word, unique identifier, synonyms]	107049
24	22 and 23	24333
25	predict*.mp. [mp=title, abstract, original title, name of substance word, subject heading word, floating sub- heading word, keyword heading word, organism supplementary concept word, protocol supplementary concept word, rare disease supplementary concept word, unique identifier, synonyms]	1278441
26	determinant*.mp. [mp=title, abstract, original title, name of substance word, subject heading word, floating sub- heading word, keyword heading word, organism supplementary concept word, protocol supplementary concept word, rare disease supplementary concept word, unique identifier, synonyms]	201153
27	22 or 24 or 25 or 26	5801700
28	decayed missing filled teeth.mp. [mp=title, abstract, original title, name of substance word, subject heading word, floating sub- heading word, keyword heading word, organism supplementary concept word, protocol supplementary concept word, rare disease supplementary concept word, unique identifier, synonyms]	231
29	community periodontal index.mp. [mp=title, abstract, original title, name of substance word, subject heading word, floating sub- heading word, keyword heading word, organism supplementary concept word, protocol supplementary concept word, rare disease supplementary concept word, unique identifier, synonyms]	627
30	community periodontal index of treatment needs.mp. [mp=title, abstract, original title, name of substance word, subject heading word, floating sub- heading word, keyword heading word, organism supplementary concept word, protocol supplementary concept word, rare disease supplementary concept word, unique identifier, synonyms]	270
31	visual plaque index.mp. [mp=title, abstract, original title, name of substance word, subject heading word, floating sub- heading word, keyword heading word, organism supplementary concept word, protocol supplementary concept word, rare disease supplementary concept word, unique identifier, synonyms]	6
32	periodontal loss of attachment.mp. [mp=title, abstract, original title, name of substance word, subject heading word, floating sub- heading word, keyword heading word, organism supplementary concept word, protocol supplementary concept word, rare disease supplementary concept word, unique identifier, synonyms]	15
33	revised oral assessment guide.mp. [mp=title, abstract, original title, name of substance word, subject heading word, floating sub- heading word, keyword heading word, organism supplementary concept word, protocol supplementary concept word, rare disease supplementary concept word, unique identifier, synonyms]	17
34	plaque index for long term care.mp. [mp=title, abstract, original title, name of substance word, subject heading word, floating sub- heading word, keyword heading word, organism supplementary concept word, protocol supplementary concept word, rare disease supplementary concept word, unique identifier, synonyms]	2
35	gingival index for long term care.mp. [mp=title, abstract, original title, name of substance word, subject heading word, floating sub- heading word, keyword heading word, organism supplementary concept word, protocol supplementary concept word, rare disease supplementary concept word, unique identifier, synonyms]	2

36	denture plaque index.mp. [mp=title, abstract, original title, name of substance word, subject heading word, floating sub- heading word, keyword heading word, organism supplementary concept word, protocol supplementary concept word, rare disease supplementary concept word, unique identifier, synonyms]	8
37	periodontal screening and recording.mp. [mp=title, abstract, original title, name of substance word, subject heading word, floating sub- heading word, keyword heading word, organism supplementary concept word, protocol supplementary concept word, rare disease supplementary concept word, unique identifier, synonyms]	38
38	denture hygiene index.mp. [mp=title, abstract, original title, name of substance word, subject heading word, floating sub- heading word, keyword heading word, organism supplementary concept word, protocol supplementary concept word, rare disease supplementary concept word, unique identifier, synonyms]	12
39	7 or 28 or 29 or 30 or 31 or 32 or 33 or 34 or 35 or 36 or 37 or 38	126194
40	oral health impact profile.mp. [mp=title, abstract, original title, name of substance word, subject heading word, floating sub- heading word, keyword heading word, organism supplementary concept word, protocol supplementary concept word, rare disease supplementary concept word, unique identifier, synonyms]	1027
41	general oral health assessment index.mp. [mp=title, abstract, original title, name of substance word, subject heading word, floating sub- heading word, keyword heading word, organism supplementary concept word, protocol supplementary concept word, rare disease supplementary concept word, unique identifier, synonyms]	65
42	geriatric oral health assessment index.mp. [mp=title, abstract, original title, name of substance word, subject heading word, floating sub- heading word, keyword heading word, organism supplementary concept word, protocol supplementary concept word, rare disease supplementary concept word, unique identifier, synonyms]	102
43	oral impact on daily performance.mp. [mp=title, abstract, original title, name of substance word, subject heading word, floating sub- heading word, keyword heading word, organism supplementary concept word, protocol supplementary concept word, rare disease supplementary concept word, unique identifier, synonyms]	44
44	9 or 40 or 41 or 42 or 43	3116
45	39 or 44	126198
46	people.mp. [mp=title, abstract, original title, name of substance word, subject heading word, floating sub- heading word, keyword heading word, organism supplementary concept word, protocol supplementary concept word, rare disease supplementary concept word, unique identifier, synonyms]	337232
47	10 and 46	20243
48	elderly.mp. [mp=title, abstract, original title, name of substance word, subject heading word, floating sub- heading word, keyword heading word, organism supplementary concept word, protocol supplementary concept word, rare disease supplementary concept word, unique identifier, synonyms]	214577
49	resident*.mp. [mp=title, abstract, original title, name of substance word, subject heading word, floating sub- heading word, keyword heading word, organism supplementary concept word, protocol supplementary concept word, rare disease supplementary concept word, unique identifier, synonyms]	152614
50	47 or 48 or 49	372541
51	21 and 27 and 45 and 50	371
52	limit 51 to year = '2008-current'	195
53	limit 52 to English language	184
54	limit 53 to abstracts	184
55	limit 54 to "all aged (65 and over)"	159

Table S5. Details of excluded studies (n=55)

	Excluded studies	Reason(s) for exclusion
1	Aguilar, M.L.; Psoter, W.J.; Montero, M.; Milord, F.; Joshipura, K.J. The quality of removable prostheses in dentate, community-dwelling elderly residing in Puerto Rico. *J. Prosthodont*. **2013**, *22*, 556-560. doi: 10.1111/jopr.12025	No older institution residents involved
2	Alcarde, A.C.B.; Bittar, T.O.; Fornazari, D.H.; Meneghim, M.C.; Ambrosano, G.M.B.; Pereira, A.C. A cross-sectional study of oral health-related quality of life of Piracicaba's elderly population. *Revista Odonto Ciência*, **2010**, *25*, 126-131. doi: 10.1590/S1980-65232010000200004	No older institution resident involved
3	Alshammari, M.; Baseer, M.A.; Ingle, N.A.; Assey, M.K.; Al Khadhari, M.A. Oral health-related quality of life among elderly people with edentulous jaws in Hafar Al-Batin Region, Saudi Arabia. *J. Int. Soc. Prevent. Communit. Dent.* **2018**, *8*, 495-502. doi: 10.4103/jispcd.JISPCD_202_18	No older institution resident involved
4	Bekiroglu, N.; Çiftçi, A.; Bayraktar, K.; Yavuz, A.; Kargul, B. Oral complaints of denture-wearing elderly people living in two nursing homes in Istanbul, Turkey. *Oral Health Dent. Manag.* **2012**, *11*(3), 107-115. www.longdom.org/open-access/oral-complaints-of-denturewearing-elderly-people-living-in-two-nursing-homes-in-istanbul-turkey-2247-2452-11-471.pdf	No oral examination/data collection.
5	Bilder, L.; Yavnai, N.; Zini, A. Oral health status among long-term hospitalized adults: a cross sectional study. *PeerJ*, **2014,** *2*, e423. doi: 10.7717/peerj.423.	Including long-term care patients as young as 19 years old; no segregation of data between younger vs. older participants
6	Bonwell, P.B.; Parsons, P.L.; Best, A.M.; Hise, S. An interprofessional educational approach to oral health care in the geriatric population. *Gerontol. Geriatr. Educ.* **2014**, *35*, 182-199. doi: 10.1080/02701960.2013.827572.	No older institution resident involved
7	Chen, X.; Clark, J.J.; Chen, H.; Naorungroj, S. Cognitive impairment, oral self-care function and dental caries severity in community-dwelling older adults. *Gerodontology*, **2015**, *32*, 53-61. doi: 10.1111/ger.12061.	No older institution resident involved
8	Chen, X.; Clark, J.J.J.; Naorungroj, S. Oral health in nursing home residents with different cognitive statuses. *Gerodontology* **2013**, *30*, 49-60. doi: 10.1111/j.1741-2358.2012.00644.x.	Did not segregate institution residents from community-dwelling elderly in data analysis.
9	Chen X.; Shuman, S.K.; Hodges, J.S.; Gatewood, L.C. Patterns of tooth loss in older adults with and without dementia: A retrospective study based on a Minnesota cohort. J. Am. Geriatr. Soc. 2010, *58*, 2300–2307. doi: 10.1111/j.1532-5415.2010.03192.x.	No older institution resident involved
10	Choi, J.S.; Yi, Y.J.; Donnelly, L.R. Oral health of older residents in care and community dwellers: nursing implications. *Int. Nurs. Rev.* **2017**, *64*, 602-609. doi: 10.1111/inr.12417	No oral examination/data collection.
11	Christensen, L.B.; Hede, B.; Nielsen, E. A cross-sectional study of oral health and oral health-related quality of life among frail elderly persons on admission to special oral health care programme in Copenhagen City, Denmark. *Gerodontology* **2012**. 29, e392-e400. doi: 10.1111/j.1741-2358.2011.00486.x.	Unclear OHRQoL assessment protocol, low quality of both clinical and OHRQoL data presented.

12	Cornejo-Ovalle, M.; Costa-de-Lima, K.; Pérez, G.; Borrell, C.; Casals-Peidro, E. Oral health care activities performed by caregivers for institutionalized elderly in Barcelona-Spain. *Med Oral Patol Oral Cir Bucal.* **2013**, *18*(4), e641-649. doi:10.4317/medoral.18767	Report focused on caregivers instead of institution residents.
13	Delwel, S.; Scherder, E.J.A; Perez, R.S.G.M.; Hertogh, C.M.P.M.; Miaer, A.B.; Lobbezoo, F. Oral function of older people with mild cognitive impairment or dementia. *J. Oral Rehabil.* **2018**, *45*, 990-997. doi: 10.1111/joor.12708	Only included older institution residents with cognitive impairment or dementia.
14	Dharamsi, S.; Jivani, K.; Dean, C.; Wyatt, C. Oral care for frail elders: knowledge, attitudes, and practices of long-term care staff. *Journal of Dental Education* **2009**, *73*, 581-588. /content/jde/73/5/581.full.pdf	Report focused on caregivers instead of institution residents.
15	Fjeld, K.G.; Mowe, M.; Eide, H.; Willumsen, T. Effect of electric toothbrush on residents' oral hygiene: a randomized clinical trial in nursing homes. *Eur J Oral Sci.* **2014**, *122*(2), 142-148. doi: 10.1111/eos.12113, *and*<br>Fjeld, K.G.; Eide, H.; Mowe, M.; Sandvik, L.; Willumsen, T. A 1-year follow-up of a randomized clinical trial with focus on manual and electric toothbrushes' effect on dental hygiene in nursing homes. *Acta Odontol. Scand*. **2018**, *76*, 257-261. doi: 10.1080/00016357.2017.1416166.	A single blinded clinical trial on electric vs. manual toothbrushing. No indication if CONSORT guideline was followed in the project; did not appear to incorporate cross-over, washout design in the project.
16	Gaszynska, E.; Szatko, F.; Godala, M.; Gaszynski, T. Oral health status, dental treatment needs, and barriers to dental care of elderly care home residents in Lodz, Poland. *Clin. Interv. Aging.* **2014**, *9*, 1637-1644. doi: 10.2147/CIA.S69790. eCollection 2014.	Study only focused on factors related to dental attendance.
17	Gerritsen, P.F.; Cune, M.S.; van der Bilt, A.; de Putter, C. Dental treatment needs in Dutch nursing homes offering integrated dental care. *Spec. Care Dentist.* **2011**, *31*(3), 95-101. doi: 10.1111/j.1754-4505.2011.00185.x.	Report focused on dental treatment need; data regarding oral health of the older institution residents at examination were not given.
18	Ghazal, T.S.; Caplan, D.J.; Cowen, H.J.; Oliveira, D. Factors related to receipt of dental care in Eastern Iowa nursing facilities. *Spec. Care Dentist.* **2015**, *35*(4), 175-181. doi: 10.1111/scd.12107.	Lacking exact details regarding dental status like caries experience, periodontal disease level, etc.
19	Gil-Montoya, J. A.; de Mello, A.L.F.; Cardenas, C.B.; Lopez, I.G. Oral health protocol for the dependent institutionalized elderly. *Geriatr. Nurs.* **2006**, *27*(2), 95-101. doi: 10.1016/j.gerinurse.2005.12.003.	The report focused on development of a oral health protocol for nursing staff.
20	Goh, C.E.; Guay, M.P.; Lim, M.Y.; Lim, S.M.; Loke, S.Y.; Toh, H.E.; Nair, R. Correlates of attitudes and perceived behavioural control towards oral care provision among trained and untrained nursing home caregivers in Singapore. *J. Clin. Nurs.* **2016**, *25*, 1624-1633. doi: 10.1111/jocn.13162.	Report focused on caregivers instead of institution residents.
21	Gopalakrishnan, A.; Kahu, E.; Jones, L.; Brunton, P. Access and barriers to oral health care for dependent elderly people living in rest homes. *Gerodontology*. **2019**, *36*, 149-155. doi: 10.1111/ger.12392.	Data regarding oral health of the older institution residents at examination was not given.

22	Hong, C.H.L.; Aung, M.M.; Kanagasabai, K.; Lim, C.A.; Liang, S.; Tan, K.S. The association between oral health status and respiratory pathogen colonization with pneumonia risk in institutionalized adults. *Int. J. Dent. Hyg.* **2018**, *16*, e96-e102. doi: 10.1111/idh.12321.	Included hospitalized subjects as young as 44 years old; did not segregate data for participants ≥60/65 years.
23	Huppertz, V. A.; van der Putten, G. J.; Halfens, R. J.; Schols, J. M.; de Groot, L. C. Association between malnutrition and oral health in Dutch nursing home residents: Results of the LPZ study. *J. Am. Med. Dir. Assoc*. **2017**, *18*, 948-954. doi: 10.1016/j.jamda.2017.05.022	No oral examination was conducted.
24	İlhan, B.; Çal, E.; Dündar, N.; Güneri, P.; Dağhan, Ş. (2015). Oral health-related quality of life among institutionalized patients after dental rehabilitation. *Geriatr. Gerontol. Int.* **2015**, *15*, 1151-1157. doi: 10.1111/ggi.12413.	A three-month prospective study on effect of oral rehabilitation upon OHRQoL change. No indication if STROBE guideline was followed in the report.
25	Jablonski, R. A.; Swecker, T.; Munro, C.; Grap, M. J.; Ligon, M. Measuring the oral health of nursing home elders. *Clin. Nurs. Res*. **2009**, *18*, 200-217. doi: 10.1177/1054773809335306	Study primarily focused on validating the feasibility of nursing and dental hygiene team in recording oral health status of nursing home residents.
26	Junges, R.; Portella, F.F.; Hugo, F.N.; Padilha, D.M.P.; Samuel, S.M.W. Caregivers' attitudes regarding oral health in a long-term care institution in Brazil. *Gerodontology* **2014**, *31*, 178-183. doi: /10.1111/ger.12019.	Report focused on caregivers instead of institution residents.
27	Kakudate, N.; Muramatsu, T.; Endoh, M.; Satomura, K.; Koseki, T.; Sato, Y.; Ito, K.; Ogasawara, T.; Nakamura, S.; Kishimoto, E.; Kashiwazaki, H.; Yamashita, Y.; Uchiyama, K.; Nishihara, T.; Kiyohara, Y.; Kakinoki, Y. Factors associated with dry mouth in dependent Japanese elderly. *Gerodontology* **2014**, *31*(1), 11-18. doi: 10.1111/j.1741-2358.2012.00685.x	Other than xerostomia, data regarding oral health of the older institution residents were not provided.
28	Kamińska-Pikiewicz, K.; Bachanek, T.; Chalas, R. The incidence of oral dryness in people over 65 years living in Lublin. *Curr. Issues Pharm. Med. Sci*. **2015**, *28*, 250-253. doi: 10.1515/cipms-2015-0082.	Other than dry mouth, data regarding oral health of the elderly surveyed were not provided.
29	Kamińska-Pikiewicz, K.; Chalas, R.; Bachanek, T. The condition of oral mucosa in the elderly (over 65 years) of Lublin. *Curr. Issues Pharm. Med. Sci.* **2017**, *30*, 39-42. doi: 10.1515/cipms-2017-0009.	Except for age and gender, did not relate poor oral mucosal condition with other factors like oral hygiene, denture status, systemic medications, etc.
30	Khabra, K K.; Compton, S.M.; Keenan, L.P. Independent older adults' perspectives on oral health. *Int. J. Dent. Hyg*. **2017**, *15*, 295-305. doi: 10.1111/idh.12259.	Qualitative research, no oral examination/data presented.
31	Khanagar, S.; Naganandini, S.; Rajanna, V.; Naik, S.; Rao, R.; Madhuniranjanswamy, M.S. Oral hygiene status of institutionalised dependent elderly in India–a cross-sectional survey. *Can. Geriatr. J.* **2015**, *18*, 51-56. doi: 10.5770/cgj.18.147	Focused in oral/denture hygiene and denture stomatitis; did not attempt to corelate oral status recorded with any data recorded.
32	Khosrozadeh, H.; Alavi, N.; Gilasi, H.; Izadi, M. Oral health-related quality of life in older people in Kashan/Iran 2015. *Nurs. Midwifery Stud*. **2017**, *6*, 182-188. doi: 10.4103/nms.nms_55_17.	No older institution resident involved
33	Lee, K.H.; Wu, B.; Plassman, B.L. Cognitive function and oral health-related quality of life in older adults. *J. Am. Geriatr. Soc*. **2013**, 61, 1602-1607. doi:10.1111/jgs.12402.	No older institution resident involved

34	Luo, J., Wu, B.; Zhao, Q.; Guo, Q.; Meng, H.; Yu, L.; Zheng, L.; Hong, Z.; Ding, D. Association between tooth loss and cognitive function among 3063 Chinese older adults: A community-based study. *PLoS ONE*, **2015**, 10(3), e0120986. https://doi.org/10.1371/journal.pone.0120986.	No older institution resident involved
35	McNally, M.; Martin-Misener, R.; McNeil, K.; Brillant, M.; Moorhouse, P.; Crowell, S.; Matthews, D.; Clovis, J. Implementing oral care practices and policy into long-term care: the brushing up on mouth care project. *J. Am Med Directors Association*, **2015**, *16*, 200-207. doi: 10.1016/j.jamda.2014.08.018	Report focused on caregivers instead of institution residents.
36	Melo, L.A.; Sousa, M.M.; Medeiros, A.K.B.; Carreiro, A.F.P.; Lima, K.C. Factors associated with negative self-perception of oral health among institutionalized elderly. *Ciência Saúde Coletiva* **2016**, *21*, 3339-3346. doi: 10.1590/1413-812320152111.08802015.	Report focused on older institution residents' self-perception of oral health; did not attempt to investigate factors associated with poor oral health conditions.
37	Naito, M.; Kato, T.; Fujii, W.; Ozeki, M.; Yokoyama, M.; Hamajima, N.; Saitoh, E. Effects of dental treatment on the quality of life and activities of daily living in institutionalized elderly in Japan. *Arch. Gerontol. Geriatr.* **2010**, *50*, 65-68. doi: 10.1016/j.archger.2009.01.013.	A six-week prospective study on effect of dental treatment upon OHRQoL, Activities of Daily Living, and Functional Independence Measure change. No indication if randomization or CONSORT guideline was followed in the report.
38	Nitschke, I.; Majdani, M.; Sobotta, B.A.; Reiber, T.; Hopfenmüller, W. Dental care of frail older people and those caring for them. *J.Clin.Nur*, **2010**, *19*, 1882-1890. doi: 10.1111/j.1365-2702.2009.02996.x.	Focused on dental attendance, no oral health data was collected.
40	Nomura, Y.; Takei, N.; Ishii, T.; Takada, K.; Amitani, Y.; Koganezawa, H.; Fukuhara, S.; Asai, K.; Uozumi, R.; Bessho, K. Factors that affect oral care outcomes for iInstitutionalized elderly. *Int. J. Dent.* **2018,** *2018*, 2478408. doi: 10.1155/2018/2478408.	A one-month prospective study on an oral health program upon body temperature of ≥37.8°C. A preliminary study, i.e. the report did not achieve the required sample size predetermined.
41	Ozkan, Y.; Özkan, M.; Kulak, Y.; Kazazoglu, E.; Arikan, A. General health, dental status and perceived dental treatment needs of an elderly population in Istanbul. *Gerodontology* **2011**, *28*, 28-36. doi: 10.1111/j.1741-2358.2010.00363.x.	A descriptive study regarding general and oral health of older institution residents. Authors did not attempt correlation analysis, if any, between oral health and other factors recorded.
42	Pessoa, D.M.D.V.; Pérez, G.; Marí-Dell'Olmo, M.; Cornejo-Ovalle, M.; Borrell, C.; Piuvezam, G.; Lima, K.C.D. Comparative study of the oral health profile of institutionalized elderly persons in Brazil and Barcelona, Spain. *Revista Brasileira de Geriatria e Gerontologia*, **2016**, *19*, 723-732. doi: 10.1590/1809-98232016019.160013.	Compared oral health of older institution residents in Brazil and Spain; no correlation analysis between oral health and various factors reported.
43	Petrović, M.; Stančič, I.; Popovac, A.; Vasović, M. Oral health of institutionalized elderly in Belgrade. *Acta Stomatol. Naissi*. **2014**, *30*, 1408-1419. doi: 10.2298/VSP150720228P.	A descriptive study regarding oral health of older institution residents. Authors did not attempt correlation analysis, if any, between oral health factors recorded.
44	Portella, F.F.; Rocha, A.W.; Haddad, D.C.; Fortes, C.B.; Hugo, F.N.; Padilha, D.M.; Samuel, S.M. Oral hygiene caregivers' educational programme improves oral health conditions in institutionalised independent and functional elderly. *Gerodontology*, **2015**, *32*, 28-34. doi: 10.1111/ger.12049.	A one-year prospective study on oral hygiene education on caregivers upon oral health and muscle strength of older institution residents. No indication if STROBE guideline was followed in the report.

45	Reis, S.C.G. B.; Marcelo, V.C.; da Silva, E.T.; Leles, C.R. Oral health of institutionalised elderly: a qualitative study of health caregivers' perceptions in Brazil. *Gerodontology*, **2011**, *28*, 69-75. doi: 10.1111/j.1741-2358.2010.00366.x.	Qualitative report focused on caregivers instead of institution residents.
46	Sampaio, N.M.; Oliveira, M.C.; Ortega, A.O.; Santos, L.B.; Alves, T.D. Temporomandibular disorders in elderly individuals: the influence of institutionalization and sociodemographic factors. *Commun. Disord. Audiol. Swallow.* **2017**, 6, *29*, e20160114. doi: 10.1590/2317-1782/20162016114.	A cross-sectional study looking into TMD in elderly individuals. No correlation analysis was conducted when data and focusing on association between TMD and resident status (institutionalized or not) as independent variable. No indication if STROBE guideline was followed in the report.
47	Schwindling, F.S.; Krisam, J.; Hassel, A.J.; Rammelsberg, P.; Zenthöfer, A. Long-term success of oral health intervention among care-dependent institutionalized seniors: Findings from a controlled clinical trial. *Community Dent. Oral Epidemiol.* **2018**, *46*, 109-117. doi: 10.1111/cdoe.12335.	A one-year prospective study on oral hygiene education on caregivers upon oral health of older institution residents. Attempted to adhere to some extent CONSORT guideline, but did not justify sample size or account how randomization was carried out, nor was blinding/masking.
48	Shaheen, S.; Kulkarni, S.; Doshi, D.; Reddy, S.; Reddy, P. Oral health status and treatment need among institutionalized elderly in India. *Indian J. Dent. Res.* **2015**, *26*, 493-499. doi: 10.4103/0970-9290.172045.	A descriptive study regarding oral health of older institution residents. Authors did not attempt correlation analysis, if any, between oral health factors recorded.
49	Smith, B.J.; Ghezzi, E.M.; Manz, M.C.; Markova, C.P. Oral healthcare access and adequacy in alternative long-term care facilities. *Spec. Care Dentist.* **2010**, *30*(3), 85-94. doi: 10.1111/j.1754-4505.2010.00132.x.	A mail survey, no oral examination was conducted nor oral health status reported.
50	Unfer, B.; Braun, K.O.; de Oliveira Ferreira, A.C.; Ruat, G.R.; Batista, A.K. Challenges and barriers to quality oral care as perceived by caregivers in long-stay institutions in Brazil. *Gerodontology*, **2012**, *29*, e324-e330. doi: 10.1111/j.1741-2358.2011.00475.x.	Qualitative report focused on caregivers instead of institution residents.
51	Veiga, N.; Diniz, L.; Pereira, C.; Amaral, O.; Coelho, I.; Pereira, J. Oral health and rehabilitation among the institutionalized elderly. *Eur. J. Soc. Behav. Sci.*, **2017**, *18*, 2353-2360. doi: 10.15405/ejsbs.208.	Report lacking details about oral health, e.g. how oral/denture hygiene was assessed nor exact data about DMFT.
52	Wang, T.F.; Chen, I.J.; Li, I. C. Associations between chewing and swallowing problems and physical and psychosocial health status of long-term care residents in Taiwan: A pilot study. *Geriatr. Nurs.* **2012**, *33*, 184-193. doi: 10.1016/j.gerinurse.2011.12.002.	No oral examination hence oral health status of the participants recorded.
53	Yoon M.N.; ickert, C.; Slaughter, S.E.; Langyel, c.; Carrier, N.; Keller, H. Oral health status of long-term care residents in Canada: results of a national cross-sectional study. *Gerodontology* **2018**, *35*, 359-364. doi: 10.1111/ger.12356.	A descriptive study regarding oral health of older institution residents. Authors did not attempt correlation analysis, if any, between oral health factors recorded.
54	Zenthöfer, A.; Meyer-Kühling, I.; Hufeland, A.L.; Schröder, J.; Cabrera, T.; Baumgart, D.; Rammelsberg, P.; Hassel, A.J. Carers' education improves oral health of older people suffering from dementia–results of an intervention study. *Clin. Interv. Aging.* **2016**, *11*, 1755-1762. doi: 10.2147/CIA.S118330.	A six-month prospective study on oral hygiene education on caregivers upon oral health of older institution residents. Attempted to adhere to CONSORT guideline, but did not

		justify sample size, randomization was not allowed by German Federal Ministry of Social Affairs; study was single blind.
55	Zenthöfer, A.; Dieke, R.; Dieke, A.; Wege, K.C.; Rammelsberg, P.; Hassel, A.J. Improving oral hygiene in the long-term care of the elderly—a RCT. *Community Dent. Oral Epidemiol.* **2013**, *41*, 261–268. doi: 10.1111/cdoe.12007.	A 12-week 'lot' randomized, single-blind, prospective study on professional cleaning, followed by dentist, old age home staff, or none oral hygiene education upon oral health of older institution residents.

Table S6. Details of all included studies listed under continent of origin in chronological order.

Country Authors (Year)	Purpose/Aim	Methodology/ Sampling method/ Sample size	Oral examination/ Instruments for oral health	Main findings on oral health	Results according to the factors
ASIA					
South Korea					
Kim et al. (2009) [43]	To assess association between chewing and oral health-related quality of life (OHRQoL) in elders (>60 years) in community or 3 of 18 long-term care facilities in Gyunnggi Province, South Korea	Cross-sectional study, cluster sampling<br><br>n = 307 community dwellers (9/25 senior centers), mean age 73.9 ± 7.3 years 197 (64.4%) female; 58 (20.4%) edentulous<br><br>n = 102 institutionalized residents, mean age 79.9 ± 7.0 years 79 (77.5%) female; 34 (43.6%) edentulous.	Calibrated dentists<br><br>Number of teeth, prosthetic status, oral health impact profile short form (OHIP-14)	• OHIP-14 were 10.6 ± 10.7, 10.3 ± 9.9 for community-dwelling or institutionalized elderly, respectively	Bivariate analysis indicated inability to pay dental bill, poor oral health status, low no. of chewable food items, poor self-reported oral health, or concern of oral health were associated with worse OHRQoL.<br><br>Did not report decayed missing or filled teeth (DMFT)<br><br>No multiple logistic regression analysis nor covariate adjustment attempted.
Iran					
Rabiei, et al. (2010) [24]	To determine the oral health status of institutionalized elderlies of 3 nursing homes in Rasht, Gilan	Cross-sectional study, convenience sample.<br><br>n = 216, ≥65 years, 163 (75.5%) female; 121 (56.0%) edentulous; 57 (26.4%)	A specialist in oral medicine<br><br>Direct visual inspection only; no details regarding	• Among the 216 residents, 91 (42.1%) had dry mouth, 54 (25.0%) had atrophic tongue, 36 (16.7%) had burning mouth, 22 (10.2%) candidiasis (with 16 or 7.4% erythematous, 6 or 2.8%	Claimed female denture wearers had higher prevalence of denture stomatitis but related data was not presented.

	Province, northwest Iran	reported no systemic disease.<br><br>71 (32.9%) had cardiovascular disease, 63 (29.6%) had hypertension and 47 (21.8%) had cerebrovascular accident, 36 (16.7%) had dementia, 31 (14.4%) had chronic obstructive pulmonary disease, 30 (13.9%) had psychiatric disease.	diagnostic criteria for oral conditions	pseudomembranous candidiasis), 15(6.9%) had traumatic ulcer, 6 (2.8%) leukoplakia (with 3 or 1.4% erythroplakia)<br>• In dentate residents (n = 95): mean standing teeth = 3.2, 49 (51.6%) had retained roots, 84 (88.4%) had caries [51 (53.7%) coronal caries, 58 (61.1%) root caries]; periodontal diseases data appeared confusing.<br>• Among edentulous residents (n = 121): 26 (21.5%) had denture stomatitis, 19 (15.7%) had denture induced hyperplasia, 13 (10.7%) had angular cheilitis, 9 (7.4%) had epulis fissuratum.	Regression analysis indicated older residents had higher gender adjusted odds for oral mucosal lesion.
Mozafari et al. (2012) [25]	To assess the oral mucosal conditions of institutionalized elderlies (≥60 years) of 5 nursing homes in Mashhad, Khorasan-e Razavi Province, northeast Iran	Cross-sectional study, convenience sample.<br><br>n = 202, mean age 79.6 ± 8.9 years, 155 (76.7%) female; institutionalized for mean 39.8 months, 78 (38.6%) had dementia.<br><br>60 (29.7%) had hypertension, 58 (28.7%) had cardiovascular disease, 50 (24.7%) had diabetes and 30 (14.9%) had cerebrovascular accident	2 calibrated specialists of oral medicine<br><br>Direct visual inspection only; no details regarding diagnostic criteria for oral conditions	• 198 (98.0%) of the surveyed residents had ≥1 oral mucosal lesion<br>• 98 (48.5%) had atrophic glossitis, 77 (38.1%) had dry mouth.<br>• 119 (58.9%) wore denture(s) for mean 19.7 years, and 109 had denture related lesion(s): 65 (54.6%) had denture stomatitis, 36 (30.2%) had epulis fissuratum, 5 (4.2%) had denture induced hyperplasia, 3 (2.5%) had angular cheilitis.<br>• Xerostomia was more prevalent in 70–79-year-old than in 60–69-year-old subjects.<br>• There was no significant difference in the prevalence of	Claimed that duration of denture wearing associate with denture related/oral mucosal lesions. No details of analysis reported.

				denture-related lesions between men and women (p> 0.05).	
India					
Kshetrimayum et al. (2013) [21]	To evaluate if OHRQoL is related to malnutrition risk in institutionalized elderly in Mysore, Karnataka, south western India	Cross-sectional study of all elderly homes, convenience sample of residents with no cognitive impairment.<br><br>n = 141, 83 (58.9%) female, mean age 72.2 ± 7.5 years, 36 (25.5%) edentulous.<br><br>Mini Nutritional Assessment Short Form (MNA-SF) administered	Calibrated examiner<br><br>DMFT Index; Geriatric Oral Health Assessment Index (GOHAI) score (continuous and dichotomized at <57: low/moderate vs. high perception of oral health)	• MNA-SF = 9.9 ± 2.4, 15.6%, 52.5%, 31.9% were malnourished, at risk of malnourish, or adequately nourished, respectively.<br>• Dentate residents had 23.5 ± 8.5 teeth,<br>• 56% residents had 20-32 standing teeth,<br>• DMFT of 11.3 ± 8.4 for dentate group; DT = 2.7 ± 3.0<br>• mean GOHAI score = 47.0 ± 9.2, 98 (69.5%) had low perception of oral health,<br>• Mean GOHAI significantly associate with mean MNA-SF	Multiple logistic regression indicated female and low/moderate perception of oral health (GOHAI <57) significantly associated with MNA-SF score.<br><br>Did not report gum/periodontal health.<br><br>Dentate residents had significant better OHRQoL than edentulous residents (49.0 ± 9.3 vs. 41.2 ± 5.8); however, they reported more discomfort in eating and swallowing, more deterrent in speaking, more sensitivities in teeth and gum, more discomfort eating in front of people and more limit in kinds and amounts of food<br><br>Female residents had worst MNF-SF scores.
Rekhi et al. (2016) [22]<br><br>(Rekhi et al. 2018 [46])[Ψ]	To investigate association between periodontal status and OHRQoL of residents from 18 elderly homes in Delhi, India	Cross-sectional study, convenience sample.<br><br>n = 500, ≥60-year, 279 (55.8%) female, 112 (22.4%) edentulous	Trained, calibrated examiner<br><br>Community Periodontal Index (CPI) for sextant-wise periodontal status: healthy (0), bleeding (1), calculus (2), moderate (3), or deep pocket (4), Loss of Attachment (LOA) of 0-3mm (0), 4-5mm (1) 6-8mm (2), 9-11mm	• 26, 117 or 131 (5.2%, 23.4% or 26.2%) residents had highest CPI of 4, 3, or 2, respectively; and 14, 114, 137 or 7 (2.8%, 22.8%, 27.8% or 1.4%) had highest LOA of 3, 2, 1 or 0, respectively. 226 (45.2%) sextants excluded<br>• MT = 15.5 ± 12.1, DT = 1.2 ± 1.7, root caries tooth = 0.6 ± 1.2<br>• 55 (11.0%) had oral mucosal lesions: 27 (5.4%) leukoplakia, 16 (3.2%) ulcerations, 11 (2.2%) candidiasis, 1 (0.2%) lichen planus	≥80-year group had more excluded sextant than 60-69-year residents<br><br>Bivariate analysis indicated residents who were female, of older age group, without formal education, poor perceived general/oral health, perceived to need dental treatment, with excluded sextants, with DT, or needing various dental prosthesis, had worst GOHAI.<br><br>No multiple logistic regression analysis nor covariate adjustment attempted.

			(3), or 12+mm (4); mobile teeth, decayed or missing teeth, oral mucosa examination; GOHAI	• mean GOHAI score = 41.6 ± 6.1	
Shivakumar et al. (2018) [23]	To assess oral health status, OHRQoL and dental care utilization of 3 elderly home residents in Satara district, Maharashtra state western India	Cross-sectional study, systematic random sampling of 3/6 elderly home, then 'random' sample of 50 residents from each.<br><br>n = 150, ≥65 years, 82 (54.7%) female, 51 (34.0%) edentulous	Dentist<br><br>CPI, DMFT, prosthetic need; GOHAI (dichotomized as per Kshetrimayum et al. 2013 [21])<br><br>Dental visit pattern	• 7, 27, 39 or 12 (4.7%, 18.0%, 26.0% or 8.0%) residents had highest CPI of 4, 3, 2 or 1, respectively;<br>• DMFT = 23.9, MT = 19.2, DT = 3.5, FT = 0.4<br>• 99/66.0% residents were with poor perception of oral health	Bivariate analysis indicated residents who needed upper or lower prosthesis were associated with poor perception of oral health.<br><br>Age and unmet dental care needs were associated with inferior/poor OHRQoL.
Hong Kong					
Tan & Lo (2014) [26]	To describe root caries situation of institutionalized elders from 21 homes in Hong Kong, China who had ≥5 teeth with exposed root; and to investigate the risk indicators for root caries in these elders.	Cross-sectional study, purposive sampling<br><br>n = 306/1,546, 233 (76.1%) female; mean age 78.8 ± 6.2 years	A trained dentist<br><br>Visible Plaque Index (VPI), Root caries index (RCI)	• Mean 14.0 ± 6.0 teeth, 53.5 exposed root surfaces; decayed root surface = 1.3 ± 1.7, filled root surface = 0.8 ± 1.9, decayed/filled root surface = 2.1 ± 2.3, modified RCI = 2.4; 67% had ≥1 decayed or filled root surface.<br>• 41%, 32%, 19%, or 7% of decayed/filled lesions at buccal, distal, mesial or lingual surfaces, respectively.	Multilevel logistic regression indicated that root surfaces with denture contact and more gingival recession had higher chance of being decayed/filled. Mesial, lingual, and distal root surfaces were less likely to be decayed/filled compared with buccal surfaces; susceptible upper anterior, upper posterior and lower posterior teeth were at higher risk (in descending order) to develop root caries then lower anteriors.
Japan					
Takeuchi et al. (2015) [27]<br><br>(Takeuchi et al. 2017 [47]) $^{\Psi}$	To investigate if posterior occlusion (natural/artificial) was associated with cognitive state and/or functional	Cross-sectional study, convenience sample<br><br>n = 234, 175 (75.2%) female; mean age 85.5 ± 7.5 years for men and 89.1 ± 6.3	Trained dentist, trained nursing home care staff<br><br>Charlson comorbidity index (CCI), Barthel	n = 234<br>• 201 (85.9%) with BMI <25<br>• 201 (85.9%) with BI <60 or were dependent for personal care	Higher total-FTUs was associated with higher MMSE scores after adjustment for demographics and teeth number.<br><br>Logistic regression analyses indicated associations between functional

	dependence of residents ≥60 years in 8 nursing home at Aso City, Kumamoto Prefecture, southern Japan	years for women; 33/14.1% (BI ≥60, i.e. functionally independent)<br><br>n = 200; 155 (77.5%) female; mean age 85.5 ± 7.8 years for men and mean age 89.4 ± 6.5 years for women; 109 (54.5%) edentulous.	Index (BI) for functional capacity or dependency of the residents; Mini-Mental State Examination (MMSE) scale for dementia.<br><br>Total-functional tooth units (FTUs)	• 18 (7.7%), 54 (23.1%) 162 (69.2%) had CCI grade 0, 1, or 2, respectively.<br>• total-FTU in functionally independent residents = 10.7 ± 3.4, functionally dependent residents = 8.7 ± 4.9.<br><br>n = 200<br>• 168 (84.0%) with BI <60<br>• mean standing teeth = 4.9 ± 7.7; 73.0% dentures wearers<br>• total-FTUs 9.3 ± 4.6.<br>• MMSE = 11.0 ± 8.6	independence and posterior teeth occlusion; more functional tooth units were associated with greater odds of independence for essential personal care; while the loss of posterior teeth occlusion was independently associated with cognitive decline.<br><br>Multivariate analyses indicated total-FTUs was positively associated with MMSE scores after adjustment against age, sex, and number of natural teeth.
Eurasian trans-continent					
Turkey					
Uludamar et al. (2011) [28]<br><br>(Evren et al. 2011 [48])$^{\Psi}$	To assess the oral health status and treatment requirements of different residential homes in Istanbul, Turkey, to study the relationship between dental caries and variables factors, and to provide guidelines for different residential homes.	Cross-sectional study, convenience sampling with cluster allocation<br><br>Uludamar et al. (2011):<br>n = 346 residents; 65-90 years, 201 (58.1%) females were 79.1 ± 7.0 years, males were 75.2 ± 8.3 years; 208 (60.1%) edentulous and 88 (25.4%) had complete denture; 135/117/94 residents were in fully financed/supported/ subsidized residential homes, respectively.<br><br>Everen et al. (2011):<br>n =269 were followed regarding denture status,	Calibrated dentists<br><br>Plaque index, DMFT, probing pocket depth (PPD) >4 mm<br><br>Denture cleanliness after plaque disclosing	• All symptom driven dental attenders with 34.3%/38.3% residents reported dental visit ≤1 year<br>• 94/269 or 34.9% never clean teeth/dentures, 51 (31.9%) removed their dentures before sleep<br>• 42.8% reported cleaning their dentures only with water; <12% brushed teeth/dentures twice a day.<br>• 59/139 or 42.4% had denture stomatitis<br>• DMFT = 25.5 ± 3.4; from 135 of the 346 cohort, DT = 3.4 ± 2.3, MT = 20.8 ± 4.5, FT = 1.2 ± 1.7<br>• From 135 of the 346 cohort, 736 teeth were examined, 59	From 346 residents study:<br>Based upon distribution, denture stomatitis appeared associated with age, income, general health, denture hygiene, or overnight denture wearing.<br><br>Apparently the surveyed residents from homes with different levels of subsidies seemed to have different levels of DMFT. The authors did not attempt post-hoc analysis to focus which group was different from the rest.<br><br>Reported no differences in prevalence of teeth with PPD >4 mm between surveyed residents from homes with different levels of subsidies

		150 (55.8%); female: 78.5 ± 7.2 years, male: 73.9 ± 8.8 years; 181 (67.3%) edentulous and 200 (74.3%) had denture; 96/80/93 residents were in fully financed/supported/subsidized residential homes, respectively.<br><br>204 (75.8%) residents had systemic disease(s): hypertension, genitourinary problems, cardiovascular and/or respiratory problems (in descending order, no details provided).		(8.0%) had moderate pockets (PPD >4mm) or above.<br><br>Evren et al (2011):<br>Out of 200 denture wearers, good denture hygiene was found in 14% of residents<br><br>Denture stomatitis was observed in 44% of residents wearing dentures	No statistically significant correlation between denture stomatitis and brushing methods and frequency.<br><br>From 269 residents study:<br>Reported significant association between residential homes and educational status, level of income, dental visiting, denture status, brushing methods, and brushing frequency; also between poor denture hygiene and the denture stomatitis.<br><br>Did not attempt multivariate analysis of data nor adjustment against confounder.
Özkan et al. (2016) [29]	To assess the oral health status of elderly residents of the Nursing Home of Aydin Social Services and to determine related risk factors and requirements in terms of oral health.	Cross-sectional study, convenience sample.<br><br>n = 114, 67 (58.8%) female; 78.5 ± 8.6 years; 54 (47.4%) edentulous; 43 (37.7%), 39 (34.2%), 24 (21.1%), 9 (7.9%) or 5 (4.4%) had neuropsychological disorders, hypertension, diabetes, kidney diseases or cancer, respectively	Dentist<br><br>CPI, LOA; DMFT, decayed root	• 5 (4.4%) visited dentist regularly, 78 (68.4%) irregular attenders<br>• 25 (21.9%) had ≥1 oral lesion, 12 (10.5%) denture stomatitis<br>• DMFT = 25.8 ± 7.5<br>• 8 (13.3%), 19 (31.7%), 23 (38.3%), 7 (11.7%), 3 (5.0%) had highest CPI of 4, 3, 2, 1, or 0, respectively; 7 (11.7%), 8 (13.3%), 18 (30.0%), 14 (23.3%), 13 (21.7%), had highest LOA of 4, 3, 2, 1, or 0, respectively.	Bivariate analysis indicated 75-84 year-group associate with poor DT, decay roots, and FT.<br><br>Did not attempt multivariate analysis of data.
EUROPE					
Lithuania					
Brukienė et al. (2011) [44]	To investigate the association between salivary flow rate,	Case-control study, convenience sample.	One examiner<br><br>Number of teeth	• Elderlies had 5.6 ± 6.9 (0-25) standing teeth	Bivariate analysis indicated negative correlation between salivary flow rate and the number of systemic diseases

	pH, buffer capacity, plaque level and health conditions plus medication in institutionalized elderlies in Vilnius, Lithuania	n = 25 'control group', 22.3 ± 2.3 years, gender unknown, all healthy, receiving no medications<br><br>n = 50 institutionalized residents, 33 (67.3%) female, mean age 82.0 ± 9.3 years; 4.7 ± 1.9 (0-9) systemic diseases, 5.3 ± 2.6 (0-10) daily medications; 31 (62.0%) edentulous, of 49 with saliva sampled.	Salivary flow rate (aspiration by saliva ejector for elderly), pH, buffer capacity<br><br>Quantitative plaque percent index: baseline plaque/plaque formation rate index	• 'Salivary flow rate' (ml/min)/pH were 0.3 ± 0.2/6.3 ± 0.7 or 0.4 ± 0.4/6.9 ± 0.5 for residents or 'control group', respectively; 38.1%/35.7%/26.2% or 0%/16.0%/84.0% of residents or 'control' had low/medium/high buffer capacity<br><br>• Baseline Plaque Index: 50.7 ± 20.9% and Plaque Formation Rate Index 39.4 ± 20.3% in 19 residents surveyed.	among the surveyed and between the number of standing teeth and the number of medications among the residents.<br><br>Baseline Plaque Index was positively correlated with the Dental Plaque Formation Rate.<br><br>Pearson correlation showed salivary buffer capacity was significantly related to salivary pH.
Spain					
Cornejo et al. (2013) [36]	To describe the oral health status and the factors associated with OHRQoL in ≥ 65 years, no cognitive impairment, institutionalized residents in 25 public social-health centers in Barcelona, Spain	Cross-sectional study, convenience sample.<br><br>n = 194, 138 (71.1%) female, 67 (34.5%) edentulous.	Dentist<br><br>CPI, DMFT, prosthetic need; GOHAI: dichotomized at <57: poor (negative) vs. good (positive).	• 135 (69.6%) had less than primary school education<br>• 15 (7.7%) attended dentists in last 12 months<br>• 10, 36, 54, 6 or 2 (5.2%, 18.5%, 27.1%, 3.1% or 1.9%) residents had highest CPI of 4, 3, 2, 1, or 0, respectively;<br>• DMFT = 22.8, MT = 20.5, DT = 2.1, FT = 0.2<br>• 145 (74.4%) were considered functional edentulous	Female residents had significantly poorer OHRQoL than males (p=0.042). They found that female residents had poorer dental and periodontal conditions, with a high prevalence of calculus and 4-5 mm pockets and a higher prevalence of edentulism than males.<br><br>Bivariate analysis indicated residents who perceived teeth, gums or denture problems, functional edentulous or needing upper denture were associated with poor GOHAI.<br><br>No multiple logistic regression analysis nor covariate adjustment attempted.
Finland					
Saarela et al. (2014) [33]	To assess ≥ 65 years service housing residents' dentition,	Cross-sectional study, convenience sample	Ward nurses evaluated dentition status interviewing,	• Mean CCI = approx. 2.9<br>• Mean BMI: 24.7-25.7<br>•	Dentition status appeared associate with age, gender, education or disability; Cox regression analysis adjusted for age,

(Saarela et al. 2014 [49])Ψ	eating habits, nutritional status (among 343 residents: energy, protein and vitamins intake, and to explore the prognostic value of dentition for mortality. Study conducted in Helsinki and Espoo, Finland	n = 1,369, 1,081 (79.0%) female; mean age 82.7 years; 708 (51.7%) edentulous; 758 (55.4%) residents had MCI or above.<br><br>Group 1: 94 (6.9%) edentulous without dentures; Group 2: 614 (44.9%) edentulous with some kind of dentures; Group 3: 661 (48.3%) with some natural teeth left.<br><br>Subgroup: n = 343; 286 (83.4%) female; mean age 83 ± 7.3 years; 162 (47.2%) edentulous; 280 (81.6%) had MCI.	observing and assessing the residents.<br><br>CCI; Clinical Dementia Rating Scale (CDR)<br><br>MNA dichotomized at <17, 17-23.5, >23.5 as malnutrition, risk for malnutrition, or good nutritional status, respectively; body mass index (BMI)	• 295 (21.5%), 286 (20.9%), 164 (12.0%), 94 (6.9%) residents with dry mouth, chewing problem, difficulty swallowing, pain in mouth, respectively; 177 (12.9%), 884 (64.6%), 308 (22.5%) were with MNA at <17, 17-23.5, or >23.5, respectively.<br><br>74 (21.6%), 220 (64.1%), 49 (14.3%) of the 343 subgroup were with MNA at <17, 17-23.5, or >23.5, respectively.	gender, comorbidity and MNA score indicated dentition status not predicting mortality.<br><br>Adjusted logistic regression analysis of the n = 343 data set indicated Group 1 predict low protein intake of <60 g/day<br><br>No DMFT recorded.<br><br>No calibration of examination accuracy by ward nurses vs. dentists.<br><br>Edentulous residents without denture had poorer physical functioning and ADL performance than those who were dentate or edentulous with or without removable denture. However, no multivariate analysis was conducted.
Germany					
Zenthöfer et al. (2014) [30]<br><br>(Zenthöfer et al. 2014 [50, 51]; 2015 [52], 2017 [53]; Klotz et al. 2017 [54])Ψ	To evaluate the association between oral health conditions, including prosthetic needs vs cognitive ability, general level of care needed, or OHRQoL of institutionalized elderlies with or without dementia in 13 homes in Baden-Württemberg, southwest Germany	Cross-sectional study, convenience sample.<br><br>n = 268, mean age 83.1 ± 9.1 years, 189 (70.5%) female; 107 (39.9%) edentulous, 136/219 (62.1%) with dementia, 3.4 ± 2.3 systemic disease, on 6.6 ± 3.5 medications.<br><br>n = 219 (68.5%) female, 136 or 62.1% with dementia, 3.4 ± 2.2 systemic disease, on 6.5 ± 3.4 medications)	Dentists<br><br>CPI of treatment needs (CPITN, or an old pre-1997 version of CPI); plaque control record; Gingival Bleeding Index (GBI), Denture Hygiene Index (DHI), Yes/No denture problem, revised oral assessment guide (ROAG): median spilt	From 93 residents pilot study:<br>• MT = 20.5 ± 8.7, DT = 0.6 ± 1.4<br>• Plaque % = 83.2 ± 20.5, DHI% = 85.1 ± 15.7; GBI% = 42.6 ± 24.1, CPITN (**unconventional data presentation**) = 3.2 ± 0.6<br><br>From 94 residents pilot study:<br>• MT = 20.3 ± 8.9<br>• after median split of ROAG score, the 'unsatisfactory' group had lower GOHAI, i.e. 48.7 ± 7.3 vs. 53.3 ± 6.2<br><br>From 92 residents pilot study:	From 94 residents pilot study: linear regression indicated compromised GOHAI significantly associated with without prosthesis rehabilitation, poor oral health and high care level, while within denture wearers only, older age, number of drugs taken, higher care levels, poor oral health, and insufficient denture condition.<br><br>From 268 residents study: Spearman correlation indicated ROAG score associated with dependency (BI) and dementia (MMSE).

		were scored for CPITN, GBI, DHI and MMSE<br><br>169 [mean age 82.9 ± 8.9 years, 114 (67.5%) female, 124 (73.4%) with dementia, 3.5 ± 2.2 systemic disease, on 6.7 ± 3.4 medications] were scored for no. of teeth, denture status (n=143), ROAG, GOHAI and MMSE.<br><br>Three pilot studies of n = 93, 94 or 92 from 4, 4 or 13 elderly homes, 60 (64.5%), 61 (64.9%) or 63 (68.5%) female, 37 (39.8%), 37 (39.4%) or 31 (33.7%) edentulous, respectively; 61.3%/74.5%/unknown with dementia, 6.6 ± 3.7/6.6 ± 3.7/unknown with systemic disease, on 7.1 ± 3.6/7.2 ± 3.8/7.3 ± 3.3 medications, respectively.	for satisfactory vs. poor oral health<br><br>German version of GOHAI dichotomized at <50: compromised vs. normal perception of oral health<br><br>MMSE, BI, Pantomime test for apraxia	• DHI = 82.6 ± 14.6%; GBI = 48.5 ± 25.9%, CPITN (unconventional data presentation) = 2.9 ± 0.7<br>• 53 (57.6%) with apraxia<br><br>Zenthöfer et al. (2014b), from 268 residents:<br>• standing teeth: 6.8 ± 8.3,<br>• 249 (92.9%) had ≥1 oral treatment need, median ROAG = 2.0; BI = 46.9 ± 30.3.<br><br>Zenthöfer et al. (2017): From 219 residents:<br>• standing teeth: 7.0 ± 8.4, edentulism data confusing (n = 78 or 92?), DHI = 82.9 ± 17.9%; GBI = 51.7 ± 28.1%, CPITN (unconventional data presentation) = 2.9 ± 0.7<br><br>Klotz, et al. (2017): From 169 residents:<br>• standing teeth: 8.3 ± 8.9, 91 (53.8%) had denture related treatment needs, ROAG = 2.3 ± 1.3; GOHAI = 49.1 ± 8.3,	From 93 residents pilot study:<br>Mean CPITN was significantly worse for the dentated residents.<br><br>From 92 residents pilot study:<br>Bivariate analysis indicated GBI % and CPITN appeared significantly worse among individuals with ideomotor apraxia than among nonapraxic participants but not for DHI%.<br><br>Linear regression showed that each identified apraxia was associated with poor oral hygiene and health.<br><br>From 219 residents study:<br>Multivariate logistic regression indicated CPITN significantly associated with female, dementia and use of coagulation inhibitors while GBI was associated with the latter and older age.<br><br>From 169 residents study:<br>The number of natural teeth <5 and edentulism without prosthesis in the residents surveyed was significant associated with low GOHAI score.
Ziebolz et al. (2017) [31]	To investigate associations between oral (dentate or edentulous) and nutritional status of 4 nursing home residents in Lower	Cross-sectional study, convenience sample.<br><br>n = 87, 68 (78.2%) female; mean age 84.0 ± 8.6 years; 41 (47.1%) edentulous, BMI = 26.2 ± 5.0 kg/m², 48 (55.2%) with dementia	Dentist<br><br>DMFT, Periodontal screening and recording (PSR) for 38 residents	• overall DMFT = 26.4 ± 3.1.<br>• residents with teeth (n=46): DMFT = 25.0 ± 3.7, DT = 2.1 ± 3.1, MT = 15.0 ± 8.3, FT = 8.0 ± 7.4<br>• 30 (78.9%) with PSR*/Periodontal screening index (PSI) score 3 and 4: needing	Multivariate analysis indicated residents at risk of malnutrition were associated with dementia, and edentulism

	Saxony, northwestern Germany		MNA, claimed dichotomize as per Saarela et al. (2014a) but considered at data analysis 8-11 as risk of malnutrition and 12-14 as normal nutrition	comprehensive periodontal treatment.<br>• Median MNA =11, 0 ‘malnourishment,’ 45 (51.7%) at risk of malnutrition of which 10 needed help with meal.	
Malta					
Santucci & Attard (2015) [35]<br><br>(Santucci & Attard 2015 [55]) Ψ	To report impact of oral health status on corresponding OHRQoL in residents of 9 state residential homes, Malta.	Cross-sectional study, convenience sample<br><br>n = 278, 221 (79.5%) female; mean age 83.5 ± 6.5 years; 56 (20.1%) current or quitted smokers, 208 (74.8%) ≥primary education, 142 (51.1%) widowed; 114 (41.0%) edentulous.	Examination at dental clinic<br><br>Modified CPI, oral mucosal lesion, prosthetic status<br><br>OHIP-14, GOHAI (243 responded), denture satisfaction questionnaire [66](169 responded)	• 29.3% (ca. n = 81?) residents reported routine dental attendance (not defined)<br>• Mean standing teeth = 13.7, 43.3% (ca. n = 120?) with caries, mean DMFT = 21.6; 22 (7.9%) had functional dentition, i.e. ≥21 teeth<br>• 64/114 edentulous residents were without denture<br>• 241 (86.7%), or 4 (1.3%) had highest CPI sextant of deep/shallow pockets; calculus, bleeding on probing or healthy, respectively<br>• Mean denture satisfaction score = 42.8; mixed reasonable satisfy and not-/un-satisfy scores<br>• 35 (12.6%) had oral mucosal lesion<br>• OHIP-14 = 3.8, GOHAI = 54.5	Spearman’s correlation indicated increased DT, MT or DMFT, without maxillary or mandibular denture was associated with better OHRQoL (OHIP-14/GOHAI); denture satisfaction was associated with denture age and denture type.
United Kingdom					
Porter et al. (2015) [37]	To assess the association between OHRQoL and oral health condition or oral symptoms of	Cross-sectional study, convenience sample.<br><br>n = 325 examined, 210 (64.6%) female, mean age	Dentist<br><br>DMFT, decayed root; self-rated oral problem	• Standing teeth = 8.9 ± 6.4, DT = 2.0 ± 1.6, D root = 2.2 ± 2.8, FT = 4.2 ± 5.2, MT = 18.0 ± 8.3<br>• 139 (68.8%) had un-rehabilitated anterior space	Adjusted multivariate logistic regression showed prevalence of oral impact (i.e. at least 1 OIDP item with a non-0 score) associated with sensitive teeth, toothache,

	residents (≥65 years) in 9 nursing homes in Islington, London, UK	82.2 ± 7.5 years; 123 (37.8%) edentulous<br><br>n = 179 responded to survey	Oral Impacts on Daily Performances (OIDP) to assess the impact of oral conditions on OHRQoL.	• 73 (40.8%), 62 (34.6%) had dry mouth or dry, sore or cracked lips, respectively. 19 (34.4%) of edentulous residents had ill-fitted denture; 23.9%, 17.1%, 15.4%, 15.4% or 10.1% of dentate residents surveyed had broken teeth, toothache, sensitive teeth, loose teeth, or bleeding gum, respectively.	loose natural teeth, bleeding gum and dry mouth.
Netherlands					
Niesten, et al. (2016) [45]<br><br>(Niesten, et al. 2017 [56])$^{\Psi}$	To investigate relationships between oral health factors and general health factors (including physical, mental, and social health domains) and OHRQoL in a care-independent and a care-dependent older population. The institutionalized elderly were from 11 residential aged care facilities in southeast Netherlands.	Case-control study, Purposive sample.<br><br>n = 126, 73 (57.9%) female, mean age 85.4 ± 7.1 years; 58 (46.0%) edentulous<br><br>109 home dwellers, 52 (47.7%) female, mean age 73.1 ± 5.4 years; 43 (39.4%) edentulous	Calibrated final year dental/dental hygiene students.<br><br>Tooth and position, caries, removable denture status<br><br>Socioeconomic status (SES), GOHAI, dental service use frequency<br><br>Gobbens' definition of frailty; Short Form health survey (SF-12: physical and mental); validated ENRICHID Social Support Index (ESSI)	• 17.5%, 50.8% of the residents surveyed had physical or mental HRQoL component ≥the population norm, respectively<br>• 26.2%, 38.9% 34.1% experienced low, medium, or high social support<br>• MT = 16.8 ± 8.2 (non-institutionalized, 10.7 ± 5.1); occluding pairs = 5.4 ± 5.0 (non-institutionalized 9.4 ± 3.5)<br><br>• 29 (42.6%) residents were caries free; 52 (41.3%) found to have no treatment need; GOHAI = 52.1 ± 6.7 (home-based control: 51.6 ± 7.4)<br><br>• 46 (36.5%), 37 (29.4%), 17 (13.5%) were classified to be of low, medium or high care-dependency, respectively.	One-way ANOVA showed higher GOHAI score from residents was associated with absence of caries, no clinical treatment needs and no reported treatment demand.<br><br>Adjusted multivariate analysis showed GOHAI score was associated with age, female gender and higher SES.<br><br>Pearson correlation indicated no correlation exist between GOHAI score of residents surveyed and any continuous variable analyzed.<br><br>Binary logistic regression indicated edentulism, difficulty in attending dentist or existence of clinical treatment need was associated with frequency or change in dental services use; while high care dependency or could not summon effort to brush was associated with change in brushing frequency. No multiple logistic

					regression analysis was attempted for the latter.
Belgium					
Janssens et al. (2017) [32]<br><br>(Janssens et al. 2017 [57]) Ψ	To describe medication use in 23 nursing homes resident and corresponding oral health; to identify various parameter that associate with poor oral health, in particular the medications related to hyposalivation. Study conducted in East and West Flanders, Belgium.	Cross-sectional study, Nonrandom sample<br><br>n =1,226; 858 (70.0%) female; mean age 83.9 ± 8.5 years; 514 (41.9%) edentulous; 81.3% had systemic disease, mean 9.0 ± 3.6 medications from 1,174 residents; 92.0%, 85.7%, 81.3%, 60.0%, or 25.5% taking medications for nervous system, alimentary tract and metabolism, cardiovascular system, blood and blood forming organs, or respiratory system, respectively.<br><br>A subgroup of n = 143 residents from 14 homes, 109 (76.2%) female; mean age 82.7 ± 7.8 years; 60.2% of low ADL level	Dentists<br><br>DMFT, PI, Dutch PSR<br><br>Activity of daily living (ADL): low, medium, or high<br><br>Anatomical Therapeutic Chemical (ATC) classification system from WHO Collaborating Centre for Drug statistics Methodology.	Among 1,226 residents:<br>• 218 (17.9%), 328 (26.9%), 675 (55.3%) had low, medium or high ADL, respectively<br>• DMFT = 27.7, DT = 1.8, MT = 24.9, FT = 1.0; retained roots = 1.1<br>• Among all prescribed medication, 49.6% or 4.5 ± 2.2 had potential hyposalivary effect<br><br>Among dentate residents (n = 712):<br>• Standing teeth = 12.3 ± 8.1, 285 (40.4%) had caries, DT = 3.0 ± 4.0 including retained roots = 1.9 ± 3.4<br>• DMFT = 24.5 ± 7.0<br>• Among residents who wore removable dentures, 36.9% needed repair, rebase or renewal.<br><br>Among 143 residents:<br>• Standing teeth = 13.6 ± 7.0, DT = 1.4 ± 2.2, FT = 2.3 ± 3.3, DMFT = 19.8 ± 6.8; retained root = 1.8 ± 3.1<br>• PI = 2.1 ± 0.7; approx. 15%, 53%, 5%, 24%, or 2% had highest Dutch PSR score of 4, 3*, 3, 2, or 1, respectively.	Among 1,226 residents:<br>Bivariate analysis showed count of medications used in dentate residents potentially induce dry mouth associated dental treatment needs/index.<br><br>Mixed-effect logistic regression analysis indicated male and age associated with proportion of DT in dentate residents while 10-13 medications used and the count of medications used potentially induce dry mouth negatively associate with the latter.<br><br>Mixed-effect logistic regression analysis indicated female, 10-13 medication used, count of medications used potentially induce dry mouth associated with higher treatment index in dentate residents while high dependency negatively associated with the latter.<br><br>General linear mixed-model analysis indicated:<br>• male and older dentate residents were associated with DT or retained roots or total treatment need<br>• female, younger dentate residents, no preferential tariff were associated with FT or restorative index<br>• male, older dentate and high dependency residents were associated with extraction need

					• female older dentate residents associate with treatment index<br><br>General linear logistic mixed analyses showed that residents with medium care dependency, increasing age and the possession of a preferential tariff were significantly associated with a higher risk of wearing a full denture.<br><br>No regression analysis attempted for interrelationship between periodontal and other socioeconomic/medical parameters of residents.
Italy					
Cocco et al. (2018) [34]	To evaluate the prevalence and severity of tooth loss in 23 nursing home residents in 5 areas (northwest, northeast, center, south and islands) of Italy.	Cross-sectional study, convenience sample.<br><br>n = 1,976, 1462 (74.0%) female; mean age 84.1 ± 9.7 years, 814 (41.2%) edentulous<br><br>BMI, MMSE, or MNA scores were collected from 1,326 (67.1%), 1,289 (65.2%), or 741 (35.5%) residents, respectively.	One examiner; not specified<br><br>Functional units (good, sufficient or insufficient for chewing; lacking clear definition), prosthetic status<br><br>SES, MMSE, BMI, MNA	• Only breakdown or per age group data available, no overall dental, SES, MMSE, BMI or MNA data presented<br>• n = 1,326; 19 (5.7%), 41 (12.2%), 90 (26.8%), 140 (41.7%), 46 (13.7%) of 336 males, 103 (10.4%), 159 (16.1%), 279 (28.2%), 313 (31.6%), 136 (13.7%) of 990 females were severely underweight, underweight, healthy weight, overweight, or obese, respectively.<br>• n = 1,289, 646 (50.1%) residents were with MCI/dementia.<br>• n = 741, 5.7%, 40.3%, 54.0% residents surveyed were with MNA at <17, 17-23.5, or >23.5, respectively.<br>• 1,688 (86.3%), 121 (6.2%), 147 (7.5%) of the 1,956 residents	Multinomial logistic regression analysis showed reduced functional dental units was associated with severe MMSE (mental state), older age group of ≥80 years, and female.<br><br>Did not collect/report DMFT/CPI data.

				surveyed had insufficient, sufficient, or good functional units for chewing, respectively.	
OCEANIA					
Australia					
Hopcraft et al. (2012) [38]<br><br>(Hopcraft et al. 2012 [58]) Ψ	To investigate oral hygiene, periodontal conditions, caries experience and edentulism in residents of 26 or 5 nursing homes in metropolitan Melbourne or rural Victoria, respectively, Victoria State, southeast Australia	Cross-sectional study, convenience sample<br><br>n = 510/1,345 surveyed; 351 (68.8%) female, women 85.7 ± 9.4 years, men 77.8 ± 13.9 years; 235 (46.1%) edentulous; 242 (47.5%) had ≥5 chronic medical conditions; 401 (78.6%) took ≥5 medications, 194 (38.0%) had dementia, 92 (18.0%) had stroke; 75 (14.7%) had diabetes (DM). Reported 273/275 dental condition.<br><br>Reported 263/275 dentate residents' periodontal conditions: n = 263, >75 years, 171 (65.0%) female; 84 (35.6%) with dementia; 37 (14.1%) had stroke; exact number of residents examined not clear.	Experienced dental epidemiologist<br><br>National Institute of Dental Research (US) protocol (teeth not dried, gross plaque removed): tooth/root surface being sound, D, F and D, F or M, (exclude when surface covered with calculus); retained root. VPI, CPI.	Dental status of 273 residents:<br>• Mean standing teeth = 14.4, DT = 2.7 with 82 (30.0%) DT = 0, and 7 (2.6%) D root, 69 (25.3%) with retained root(s)<br><br>Periodontal status of 263 residents:<br>• Only 25% residents had dental visit in past 2 years,<br>• <1/3 residents reported cleaning their teeth ≥2×/day;<br>• 50% of residents reported cleaning their teeth 1×/day,<br>• >25% residents had plaque covering >1/3 of at least one index tooth (upper first and second molars, upper right central incisor; lower first and second molars, lower left central incisor),<br>• All residents had visible plaque on any one index tooth<br>• Periodontal health was extremely poor;<br>• >50% of residents had calculus	Male gender or residents ≤65 years old had significantly more DT and fewer FT; residents claimed that needing dental treatment had more DT; those who attended dentists ≤1 year had higher DMFT; residents attended dentist for check-up had more FT; dentated DM residents had less standing teeth, less FT and more DT; no association detectable between DT and number of medications taken nor number of chronic medical conditions; no post-hoc test nor regression analysis attempted.<br><br>Female residents had increased visual plaque; 75-84 years group had increased visual plaque and periodontal disease<br><br>Logistic regression analysis indicated CPI score 3 or above (i.e. periodontal pocket 3.5-5.5 mm [35.6% participants] or periodontal pocket >6 mm [10.2%]) in any one sextant of a participant was associated with inadequate or poor plaque control, >9 teeth present, male gender and aged 75-84 years; no covariate adjustment attempted.<br><br>Residents who required total assistance with oral hygiene had more decayed teeth and fewer filled teeth.

Philip et al. (2012) [39]<br><br>(Philip et al. 2012 [59]) Ψ	To investigate oral hygiene, gum conditions and caries experience of institutionalized, aged, dentate residents in Perth, Western Australia, with or without dementia	Cross-sectional study, convenience sample.<br><br>n = 205, mean age 85.0 ± 9.8 year; 84/186 (45.2%) with dementia; 193 with modified Activities of Daily Oral Health (mADOH) score measurement	Dentist<br><br>Full mouth examination: O'Leary's plaque score (Pl%); visual detection of gingival redness (categories of minimal, light, moderate or heavy inflammation).<br><br>DMFT, retained root	• 21% residents were mADOH able, 22% as partially disabled, and 51% disabled<br>• DMFT ≈ 26, DT ≈ 3, MT ≈ 18, FT ≈ 5; D root ≈ 0.05, retained root ≈ 1.3.	Poor mADOH associated with more DT or retained roots; did not attempt regression analysis e.g. between Pl% or DMFT and other variables.<br><br>Higher full mouth means Pl% associated with dementia, partially disabled or disabled or assisted with oral care<br><br>Gingival inflammation associated with dementia, partially disabled or disabled or assisted with oral care<br><br>No multiple regression, no covariate adjustment attempted.
NORTH AMERICA					
Canada					
Kotzer et al. (2012) [40]<br><br>(Matthews et al. 2012 [60]) Ψ	To investigate oral health status and OHRQoL of pre-seniors (45-64 years), seniors (≥65 years), and residents in 31 (4 private, 28 public; 15 rural, 16 urban) long term care (LTC) facilities in urban and rural Nova Scotia, maritime province, eastern Canada.	Cross-sectional study, random sampling<br><br>*Oral health survey:*<br>LTC residents: n = 335, 248 (74.0%) female; mean age 80.8 ± 11.6 years; 137 (40.9%) edentulous; 112/329 (34.0%) perceived fair/poor general health.<br><br>*OHRQoL study:*<br>Community dwellers: pre-seniors n = 629, seniors n = 501, 711 (62.9%) female<br><br>pre-senior LTC residents n = 33, elderly LTC residents n = 297; 246 (74.5%) female	6 calibrated dentists<br><br>Modified WHO protocol: mucosal and denture status, DMFT, gingival index (GI), debris index, calculus index, PPD, probing attachment level (PAL)<br><br>OHIP-14	• 244 (75.3%) LTC residents reported dental visit <1×/year; 64.6%/28.6%/10.5%/6.5%/1.5%/ 1.8% reported oral healthcare provider as none, dentist, dental hygienist, denturist, physician, or others, respectively.<br>• 155 (48.7%) residents brush <2×/day, 158 (83.2%) of 190 dentate residents did not use floss daily.<br>• 79/324 (24.4%) perceived fair/poor oral health<br>• 37.3%/8.3%/8.0%/8.0% complained of dry mouth, sensitivity, bleeding on brushing, or pain around temporomandibular joint (TMJ), respectively.	Logistic regression indicated that perceived need for oral treatment and smoking years were associated with ≥1 D/F roots, while debris index ≥2 and smoking years were associated with ≥1 D crown, or residents ≥65 years; brush <1×/day, and smoking years were associated with DMFT in dentate residents.<br><br>Logistic regression indicated that residents ≥75 year-old, from urban homes, and without fairly/very often OHIP-14 impact were associated with usage of complete upper and lower dentures all the time.<br><br>Bivariate analysis indicated that LTC residents who reported one or more

	• 138 (41.2%) residents had some sort of oral mucosa conditions (or edentulous alveolar ridge resorption): 14.0%/12.2%/7.2%/6.0%/4.2%/ 3.3%/3.03 had extreme alveolar ridge atrophy, denture stomatitis, glossitis, ulcer, white patches, angular cheilitis, residual ridge fibrosis, respectively.<br>• DMFT = 23.6 ± 5.3, DT = 0.8 ± 1.8, MT = 19.7 ± 9.0, FT = 3.1 ± 4.9<br>In 198 dentate LTC residents<br>• DT = 1.4 ± 2.2, FT = 5.2 ± 5.4; D root = 1.4 ± 2.6, F root = 1.1 ± 2.1; 51.0% had untreated coronal caries, 44.4% had untreated root caries<br>• Debris index = 1.4 ± 0.7, calculus index = 1.1 ± 0.8; 65.6% surveyed had GI ≥2 at ≥1 site; 36.2% surveyed had PPD ≥4 mm at ≥1 site; 66.7% surveyed had PAL ≥ 4 mm at ≥1 site.<br>• 34.0%/21.0% residents perceived fair/poor general health or quality of life, respectively.<br>• OHIP-14 scores: community dwellers = 5.6 ± 7.6, LTC residents = 5.6 ± 9.6<br>• OHIP-14 scores in seniors: community dwellers = 4.75±6.92, LTC residents = 5.71 ±9.80.	impacts "fairly often" or 'very often' were associated with high school education or lower, had oral pain, perceive their general health, mouth health and quality of life to be fair or poor, or dissatisfied with their teeth or dentures.<br><br>Logistic regression indicated that LTC residents having ≤high school education, with fair or poor perceived mouth health were more likely to report an OHIP-14 response of 'fairly often' or 'very often'.
USA		

Zimmerman et al. (2017) [41]	To characterize oral hygiene status of 14 nursing homes in North Carolina (11 profit making, with high pneumonia re-hospitalization rate) residents and examine risk that might correlate with information in the minimum data set.	Cross-sectional study, convenience sample.<br><br>n = 506, 361 (71.3%) female, 79.2 ± 12.8 years, 324 (64.0%) white, 148 (29.2%) black, in nursing home for 2.5 ± 2.3 years; 106 (20.9%) edentulous; 259 (51.2%) dementia or Alzheimer's disease, 86 (17.0%) received antipsychotic or antianxiety medications last week.	Dental hygienist<br><br>Plaque index for long term care (PI-LTC, n = 400), GI for LTC (GI-LTC, n =397), denture plaque index (DPI, n = 176)	• PI-LTC = 1.7 ± 0.8 (out of 3); GI-LTC = 1.5 ± 0.9 (out of 4), DPI = 2.2 ± 1.2 (out of 4),<br>• lower denture evidencing better hygiene, especially on the facial (exposed) surface	Bivariate analysis indicated PI-LTC associated with profit making nursing homes, less proportion of residents under Medicaid, dental hygienist visit in last quarter, shorter length of stay, with dementia or above, or residents currently on hospice; while GI-LTC associated with shorter length of stay, with dementia or above, or residents currently on hospice. DPI associated with lower quality of nursing home rating, private payment, with dementia or above.<br><br>Cognitive problem was significantly related to all measures.<br><br>Did not collect/report DMFT/CPI data<br>No DMFT/CPI data<br><br>Did not attempt multivariate data analysis.
SOUTH AMERICA					
Brazil					
Piuvezam & de Lima (2012) [42]<br><br>(Piuvezam & de Lima, 2013 [61]; de Medeiro et al. 2019 [62]) $^{\Psi}$	To identify self-perceived oral health status, factors associated with missing teeth or TMJ alterations in institutionalized elderly in 2 cities each in 11 medium and large municipalities representing North, Northeast, South,	Cross-sectional study, convenience sample.<br><br>n = 1,192, 638 (53.5%) female, 76.3 ± 9.8 year; 605 (50.8%) with impaired cognitive conditions; the rest, or n = 587 examined, 285 (48.6%) female, 75.0 ± 9.5 years, 320 (54.5%) edentulous; n = 979 for TMJ examination, 677 (58.4%) edentulous.	Dentists, n = 5, with intra and inter-examiner calibration<br><br>CPI, LOA, DMFT; prosthetic need, TMJ examination; GOHAI score, binary approach adopted: dichotomized at ≤30: negative vs. positive perception of oral health	• 127/583 (21.8%) residents had dental visit in past 12 months<br>• 25, 49, 109, 4 or 22 (4.3%, 8.3%, 18.6%, 0.7% or 3.7% of total) had highest CPI of 4, 3, 2, 1 or 0, respectively; and 16, 31, 58, 57 or 47 (2.7%, 5.3%, 9.9%, 9.7% or 8.0% of total) had highest LOA of 4, 3, 2, 1 or 0, respectively. 378/64.4% sextants excluded<br>• DMFT = 28.8 ± 5.5 (> 28, i.e. **third molars not excluded**); from	DMFT appeared incorrect, i.e. **3rd molars** perhaps **not excluded** making final DMFT >28.<br><br>Adjusted multivariate analysis indicated males, residents last dental visited >1 year, lack of private health assistance, or at non-South Brazil regions were associated with higher rehabilitation needs; while residents self-perceived fair gum, teeth or prosthesis had more extraction needs; and, females, residents living independently, self-perceived poor

	Southeast, and Midwest Brazil.<br>Total of 36 long-stay institutions for the elderly (LSIE) were surveyed.		the 1,192 cohort, DT = 1.2 ± 6.7, FT = 0.3 ± 1.2<br>• 454 (39.2%)/245 (21.2%) wearing upper/lower denture<br>• 709 (61.2%)/908 (78.4%) need some forms of upper/lower denture<br>• 179/15.5% had TMJ alternation<br>• median GOHAI score = 33	gum, teeth or prosthesis or needing upper dentures were associated with more TMJ alterations.<br><br>Adjusted multivariate analysis indicated residents who visited dentist in last year, had gum problems or self-perceived poor gum, teeth or prosthesis were associated with poor GOHAI scores.

[Ψ]indicates the article(s) was/were referred because the articles(s) shared the same sample group with the main included study and reported relevant information that could not be retrieved in the main study.
